# Environmental influence of gaseous emissions from self-heating coal waste dumps in Silesia, Poland

**DOI:** 10.1007/s10653-018-0153-5

**Published:** 2018-07-24

**Authors:** Monika Fabiańska, Justyna Ciesielczuk, Ádám Nádudvari, Magdalena Misz-Kennan, Adam Kowalski, Łukasz Kruszewski

**Affiliations:** 1 Faculty of Earth Sciences, University of Silesia, 60 Będzińska Street, 41-200 Sosnowiec, Poland; 2 Institute for Ecology of Industrial Areas, 6 Kossuth Street, 40-844 Katowice, Poland; 3 Faculty of Geology, Geophysics and Environmental Protection, AGH University of Science and Technology, 30 Mickiewicza Avenue, 30-059 Cracow, Poland; 4 Institute of Geological Sciences, Polish Academy of Sciences (ING PAN), 51/55 Twarda Street, 00-818 Warsaw, Poland

**Keywords:** Coal wastes, Self-heating, Gas emission, Volatile organic compounds, Greenhouse gases

## Abstract

Gaseous emissions from seven self-heating coal waste dumps in two large coal mining basins, Upper and Lower Silesia (Poland), were investigated by gas chromatography (GC-FID/TCD), and the results were correlated with on-site thermal activity, stage of self-heating as assessed by thermal mapping, efflorescences, and surface and subsurface temperatures. Though typical gases at sites without thermal activity are dominated by atmospheric nitrogen and oxygen, methane and carbon dioxide are present in concentrations that many times exceed atmospheric values. On average, their concentrations are 42.7–7160 ppm, respectively. These are levels considered harmful to health and show that coal waste fire can be dangerous for some years after extinction. At thermally active sites, concentrations of CH_4_ and CO_2_ are much higher and reach 5640–51,976 ppm (aver.), respectively. A good substrate–product correlation between CO_2_ and CH_4_ concentrations indicates rapid in-dump CH_4_ oxidation with only insignificant amounts of CO formed. Other gas components include hydrogen, and C_3_–C_6_ saturated and unsaturated hydrocarbons. Decreasing oxygen content in the gases is temperature-dependent, and O_2_ removal rapidly increased at > 70 °C. Emission differences between both basins are minor and most probably reflect the higher maturity of coal waste organic matter in the Lower Silesia dumps causing its higher resistance to temperature, or/and a higher degree of overburning there.

**Electronic supplementary material:**

The online version of this article (10.1007/s10653-018-0153-5) contains supplementary material, which is available to authorized users.

## Introduction

Coal waste dumps, a common landscape feature in coal mining regions, are a potential source of hazardous substances emitted to the atmosphere and leached to surface and ground waters (e.g., Grossman et al. 1994; Stracher and Taylor 2004; Finkelman 2004; Pone et al. 2007; Querol et al. 2008; Carras et al. 2009; Hower et al. 2009; O’Keefe et al. 2010; Skręt et al. 2010). Negative influences on the environment are increased in the case of dumps where self-heating, or even open fire, occurs; these processes release a wide variety of gases and water-soluble inorganic and organic compounds.

Recent research has focused on the reasons for self-heating, its prevention and fire extinction (e.g., Krishnaswamy et al. 1996a, b; Kaymakçi and Didari 2002; Singh et al. 2007; Querol et al. 2011). However, a developing awareness of the environmental impact has increased attention on self-heating products and their polluting potential. The gaseous products are of particular interest due to their toxicity, carcinogenicity, and greenhouse significance, even though it is very difficult to reliably assess total quantities expelled in any given instance (e.g., Yan et al. 2003; Stracher and Taylor 2004; Finkelman 2004; Younger 2004; Kim 2007). As gases and volatile organic compounds (VOCs) are the first substances released during the initial low-temperature stage, they can be used to monitor the thermal state of coal waste dumps (Tabor 2002; Xie et al. 2011). As these dumps are commonly located in highly populated industrial regions, they should be deemed major environmental and health hazards. Though persistent odors and dust are an obvious problem for nearby residents, the most harmful emissions (e.g., CO, CO_2_, and monoaromatic hydrocarbons) are odorless. The gases also contain NO_x_, NH_3_, SO_x_, and H_2_S from the thermal decomposition of sulfide minerals, HCl, light aliphatic compounds up to C_10_, aromatic compounds such as benzene and its alkyl derivatives, styrene, alcohols, PAHs, and heavy metals, e.g., Hg, As, Pb, and Se (Stracher and Taylor 2004; Pone et al. 2007; O’Keefe et al. 2010; Querol et al. 2011). Halogenated organic compounds, e.g., CH_3_Cl, may form during the thermal decay of clay minerals and subsequent hydrohalogen reactions with organic matter (Davidi et al. 1995; Fabiańska et al. 2013). Sulfur, oxygen, and nitrogen heterocyclic compounds such as furane, thiophene, and pyridine derivatives have also been noted (Ribeiro et al. 2010).

The major compound emitted during self-heating is CO_2_, accompanied by CO and light organic compounds. These derive partially from gaseous compounds trapped in organic matter pores and partly from pyrolysis, depending on the temperature range, coal rank, and oxygen availability (Davidi et al. 1995; Younger 2004; Querol et al. 2008). It is extremely difficult to assess the scale of emission of the two main greenhouse gases, CO_2_ and CH_4_, in the field due to spatial and temporal emission variability, mixing of gas with air, the large volume of coal wastes, and the influence of weather (Litschke 2005). Research on emission fluxes from Australian coal waste dumps has shown CO_2_ emission from 12 to 8200 kg CO_2_/m^2^ per year (Carras et al. 2009). Liu et al. (1998) estimated that the combustion of one tonne of coal waste can generate 99.7 kg CO, 0.61 kg H_2_S, 0.03 kg NO_x_, 0.84 kg SO_2_, and 0.45 kg smoke.

The aims of our study were (a) to examine the variability in occurrence and distribution of the main gas components emitted from self-heating dumps in Upper and Lower Silesia, (b) to establish whether differences in gas distributions are related to thermal stage, (c) to compare the activity and dynamics of self-heating in the two basins, and (d) to assess levels of hazardous gas emissions in both. Preliminary research on gas compositions performed in Upper Silesia (Fabiańska et al. 2013) aided selection of appropriate components.

## Materials and methods

### Coal waste dumps

#### Wełnowiec dump (Upper Silesia)

**Fig. 1:**
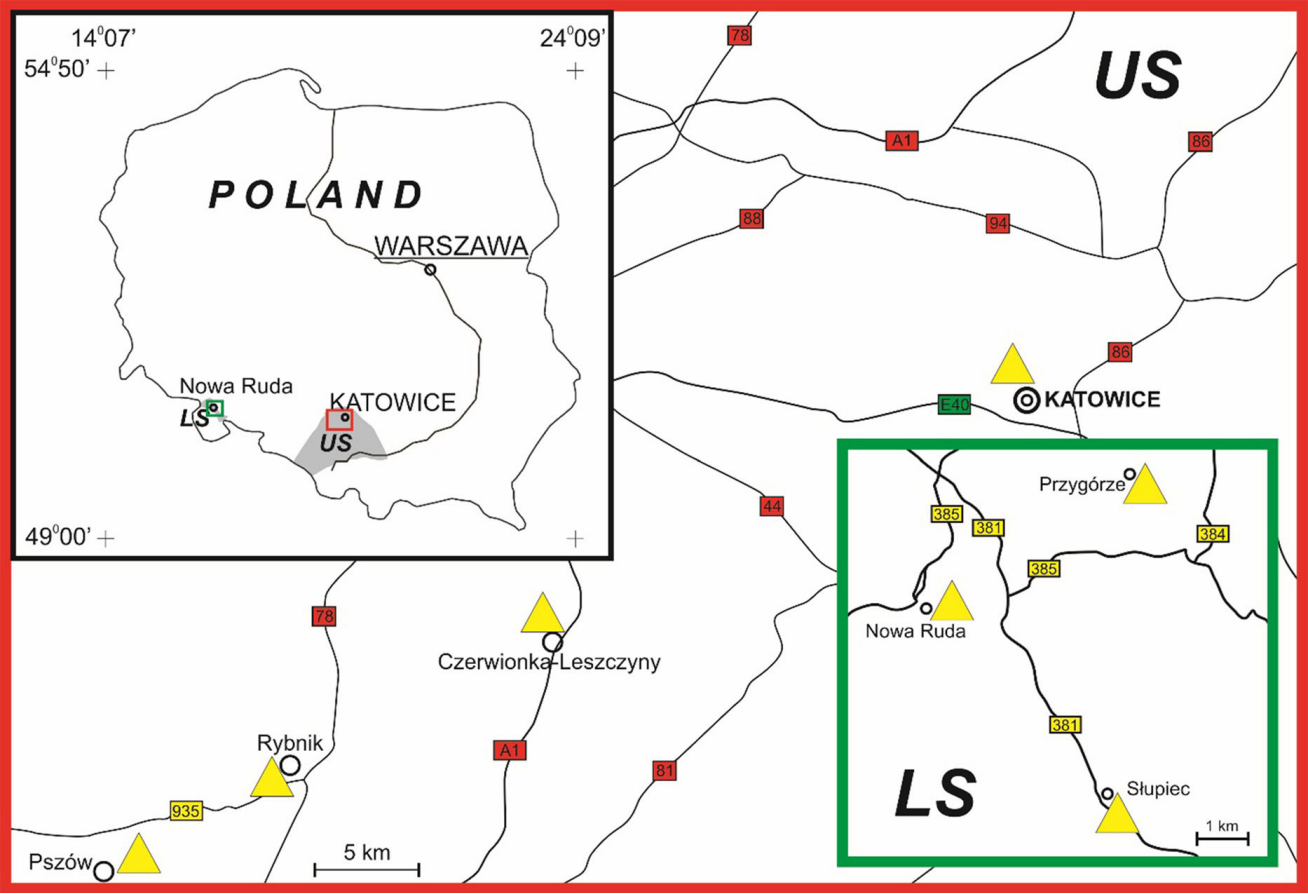
Location of coal waste dumps from which gas was collected; LS (Lower Silesia) and US (Upper Silesia)

The Wełnowiec dump in Katowice operated as a municipal waste dump from 1991 to 1996 (Figs. 1 and S1a). The dump area is 16 ha and its capacity is 1.6 mln t. About 22.5% of the deposited waste is gangue rock from coal mining (sandstones, carbonates, siltstones, and clays), 21.5% is municipal waste, and 40% is rubble. A reclamation project was designed to involve a multilayered barrier system comprising layers of soil, coal waste with < 5% organic matter, gravel, sand, and clay liners. In fact, a much thicker layer of coal waste with much higher carbon contents was deposited in a random fashion with no evidence of any barriers. As a result, self-heating started. Temperatures reached ca 700 °C (Ciesielczuk et al. 2013, 2015). In recent times, heating essentially ceased after the application of various fire-extinguishing methods, culminating with the deposition of waste from a sewage cleaning plant.

#### Rymer cones (Upper Silesia)

The Rymer Cones dump (Fig. 1) was linked to coal exploitation in the Rymer Coal Mine from 1858 to 2011 (Frużyński 2012). Today, the dump covers the area of 0.13 km^2^, its height is > 300 m a.s.l., and its capacity is 2.4 × 10^6^ m^3^ (Barosz 2003). Coal waste was loosely deposited in three cones loosely without compaction and without ground sealing. Over time, self-heating has altered most of the waste. To halt the heating, the dump was redeveloped in 1994–1999 and encased with waste from current mining (Tabor 2002; Barosz 2003). In the process, two cones were dismantled and combined, and a plateau formed on top on which fly ash pulp was deposited. The remaining cone was covered with concrete panels and fly ash to block air access and, thereby, to stop the self-heating. These efforts failed, and self-heating restarted and intensified. Currently, activity appears to be slowly diminishing with heating confined mostly to the eastern slope.

#### Anna dump (Upper Silesia)

The dump stores waste from the Ruch Anna Coal Mine opened in 1954 in Pszów (Fig. 1). A single cone covers an area of 0.43 km^2^, the oldest part (~ 0.20 km^2^) of which and is ~ 50 m high. Planned capacity was > 3 × 10^6^ m^3^ (Barosz 2003). Exploitation for road building enabled oxygen access, and intensified self-heating hindered further exploitation. Toxic fumes and odors are now a problem in Pszów (Misz-Kennan et al. 2013). In 2015, the cone was flattened. Any current heating is reflected in puddles of tar, cracks, gas vents, and salamoniac crusts.

#### Czerwionka-Leszczyny dump (Upper Silesia)

The mostly forested dump located in Czerwionka-Leszczyny (Fig. 1) consists of three cones, the highest of which is ~ 100 m high. On its top, intense surface pseudo-fumarolic activity is associated with surface gas vents surrounded by sulfate crusts (Parafiniuk and Kruszewski 2010). Some tar puddles reflect heating under pyrolytic conditions that caused thermal cracking of the coal waste organic matter macromolecule. The tar migrated to accumulate on relatively cold coal waste surfaces (Nádudvari and Fabiańska 2016). The thermal activity, extant for more than 30–40 years, is waning, and burnt-out material is evident in parts of the dump (Nádudvari 2014).

#### Nowa Ruda, Słupiec and Przygórze dumps (Lower Silesia)

Hard coal exploitation began in Lower Silesia in the 1400s, especially around Wałbrzych and Nowa Ruda (Fig. 1). Several mines operated there in the 1900s. Mining ceased in 2000 when the mines became unprofitable (Frużyński 2012). A few hundred years of mining left coal waste dumps in which self-heating lasted for many tens of years. Despite several attempts to halt them, fires still occur today. The dumps contain waste that is commonly completely altered.

The coal waste dump in Nowa Ruda was heaped up after 1945. Covering an area of ca 0.4 × 0.5 km, it is < 110 m high (523 m a.s.l.) and contains 10.2 mln tonnes of waste (Borzęcki and Marek 2013). At present, thermal activity is observed at its top and on the slope nearby. Elsewhere, snow cover remains in winter, unlike as in the past. More thermally active sites occur in the Słupiec dump (Fig. S1b). The dump in Przygórze is now cool though tonnes of overburnt coal waste attest to intense past activity.

### Thermal activity of coal waste dumps

How coal waste dumps are affected by fire that depends on a variety of factors, e.g., fire duration, oxygen access, volume of burning waste, the nature of organic material, its content, and petrographic composition. Gas sampling sites were chosen where signs of thermal activity were evident, namely open fire, smoke, odors, charred vents, efflorescences, a lack of vegetation, or the presence of moss or mullein (*Verbascum L.*; Figs. S2 and S3). The thermal activity stage was established mainly on field observations and temperature measurements.

Thermal sites classed as ‘ongoing’ show increased surface and subsurface temperatures, visible smoke, mineral efflorescences, and tar seepage. Thirty-nine gas samples were collected from such at five dumps. ‘Initial’ thermal activity was recognized only at the Wełnowiec dump (4 samples) in places where fire was beginning to encroach on cool coal waste. Here, temperatures are high, smoke, and odors noticeable, and blooming organic efflorescences prominent. Nine samples are from sites of ‘waning’ thermal activity marked by lower temperatures and a lack of efflorescences. In addition, five samples are from sites with no current thermal activity; these were never touched by fire or past activity had ceased.

### Thermal mapping

Thermal maps help to reveal the self-heating history of coal waste dumps. They can aid the location of current hot spots, their migration paths, and variations in intensity with time. Regretfully, such archival data are rarely available for dumps. For this study, a series of Landsat 5, 7, and 8 images with snow covering was used. The thermal mapping procedure used is detailed in Nádudvari (2014). Hot spots on the dumps may appear as high-temperature surface anomalies. Extended observation enables recognition of persistent heat sources due to self-heating, and their migration, intensification, and disappearance if they are hot enough to detect with satellite sensors (Tetzlaff 2004; Zhang and Kuenzer 2007; Prakash et al. 2011; Nádudvari 2014).

**Fig. 2:**
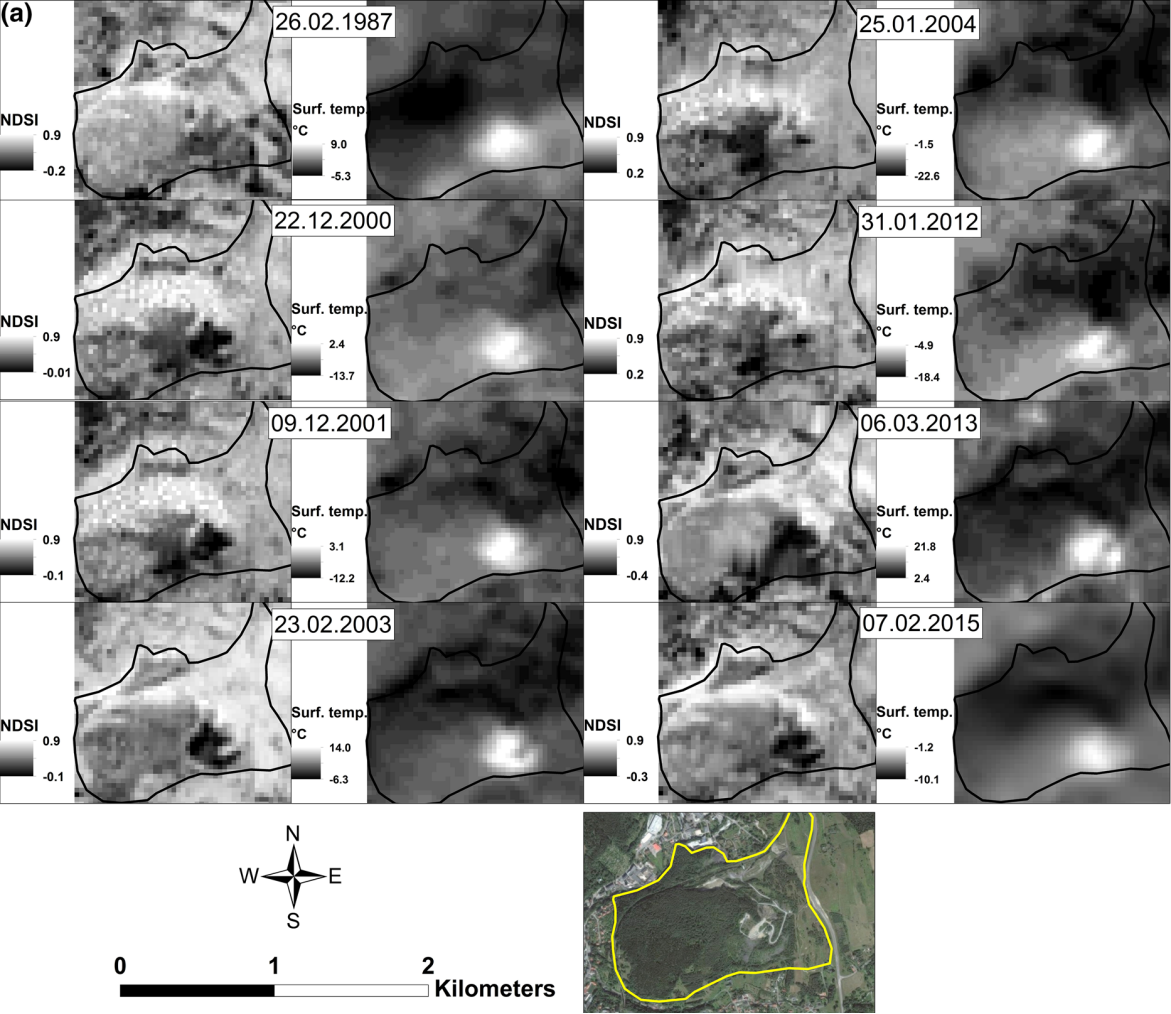

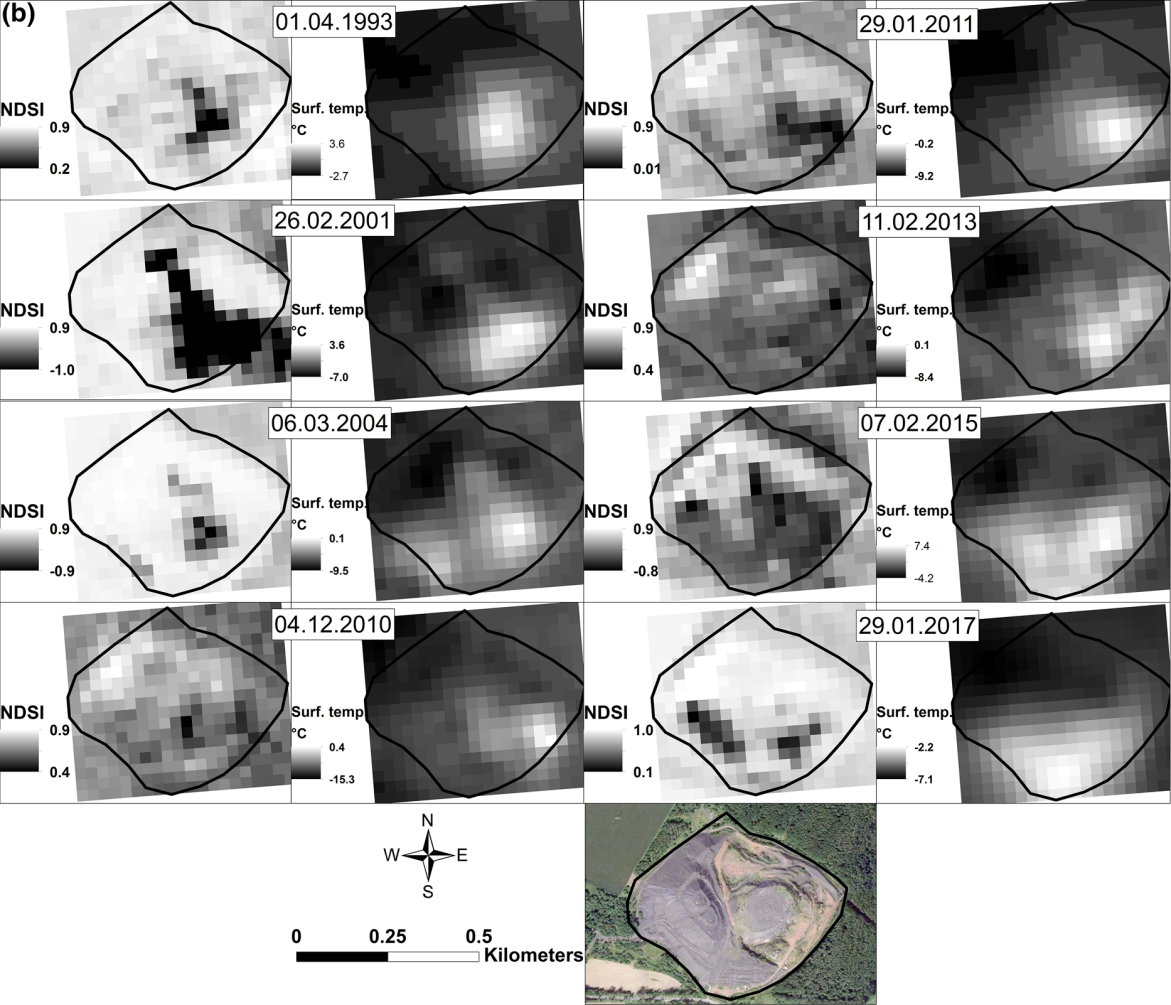
Development of thermal zones as revealed by thermal mapping. **a** Nowa Ruda dump (Lower Silesia), **b** Anna dump (Upper Silesia)

In general, most coal waste hot spots where intensive fire is present can be detected when *T* values are 6–14 °C higher than background surface temperatures (Table S1). Cold and frosty weather can induce a marked decrease in hot-spot surface temperatures (Fig. 2). Where intense fires take place, the lack of snow covering is indicated by NDSI (Normalized Difference Snow Index) values < 0; abundant snow is indicated by values > 0.5 (Nádudvari 2014). The resolution of the thermal bands of the applied Landsat series varies from 60 to 120 m (Landsat TM–120 m, Landsat ETM + − 60 m, Landsat 8–100 m) where pixel size is reduced to 30 m (https://landsat.usgs.gov/what-are-band-designations-landsat-satellites). Thus, fires falling below these resolutions, or have low surface temperatures, evade detection.

### Lower Silesian coal waste dumps

Eight Landsat images from 1987 to 2015 (NDSI index values and melted snow) clearly show continuous thermal activity during that period in the coal waste dumps in Nowa Ruda (Fig. 2a) and Słupiec. On these dumps, self-heating resulted in elevated temperatures in 1987, 2000, 2001, and 2003 despite mostly sub-zero background temperatures. Since 1987, hot-spot migration is evident in Nowa Ruda, as is the appearance of a new burning site within the constantly active heating zone there. Generally, in all dumps, the intensity of self-heating is waning. The Przygórze dump showed no intense activity during 1987–2015.

### Upper Silesian coal waste dumps

Eight Landsat images from 1993 to 2017 reveal that thermal activity of varying intensity has been constant over that period on the Rymer dump. The self-heating center moved toward the eastern side of the dump and divided into two main hot spots. However, the fires are not characterized by high-temperature anomalies. The relatively low thermal activity here may be due to the covering of concrete panels and a deeper siting of the hot spots. On Wełnowiec dump, intense thermal processes are difficult to distinguish; the hot spots fall below the satellite sensitivity limits. In the Czerwionka-Leszczyny dump, the fire at the top of the highest cone has been waning since the early 1990s (Nádudvari 2014). The Anna dump (Fig. 2b) showed intensive thermal activity in 2001, 2004, and 2010, despite frosty ambient temperatures; the increased activity was a response to exploitation. Today, the heating is less. Though thermal maps for 2017 do not show strong thermal anomalies, possibly reflecting the low resolution of the thermal band of Landsat 8, a lack of snow covering on the southeast side of the dump indicates that burning continues. The lack of snow covering on southwest side is related to deposition of new waste material.

### Gas sampling

Fifty-seven gas samples collected from the seven dumps (Table S2) include 12 from Wełnowiec, 10 from Rymer Cones, 5 from the Anna dump and 5 from Czerwionka-Leszczyny in the Upper Silesian Coal Basin, and 21 from Słupiec, 3 from Nowa Ruda, and 1 from Przygórze in the Lower Silesian Coal Basin (Figs. S2 and S3). Numbers of samples reflected numbers of active sites and their intensity. Only at the Wełnowiec dump was it possible to clearly distinguish all three stages of thermal activity. Two sites (W2 and W3) relate to the initial stage, one (W4) to ongoing activity, and two (W5 and W6) to waning fire (Figs. S1a and S2). Sites W1, W7, and W8 that had seen fire in the past were inactive on the day of sampling (13.01.2014). Rymer Cones was sampled on 14.04.2011 (R1a, R2, R4a, R5a, and R6a) and, four years later, on 16.03.2015 (R1b, R3, R4b, R5b, and R6b) at sites of ongoing thermal activity. Samples from the Anna dump and Czerwionka-Leszczyny were collected on 12.11.2016 from sites of thermal activity that had been waning since September 2016 at least. Sampling took place at Słupiec on 13.12.2013 and at Nowa Ruda and Przygórze on 28.5.2014 (Figs. S1b and S3). At Słupiec, ten thermally active sites from the top and slopes of the dump were sampled at varying depths (S1–S10) and a reference sample (S11) was collected at 1 m depth at a site where thermal activity had never been noted. Though the Nowa Ruda and Przygórze dumps had been very active in the past, at Nowa Ruda, only two sites are still active (N2 and N3) and any in the Przygórze dump has ceased (P1).

Samples (100 cm^3^) were collected a few centimeters subsurface and deeper (< 1.5 m) using syringe samplers. A 1.5-m steel pipe protected by a clinch was hammered as deeply as possible into vents or heated spots. The clinch was removed, and the gas was collected using a plastic pipe with an attached syringe fixed to the steel pipe. The clinches were abandoned.

### Temperature measurements in situ

Temperature was measured using a pyrometer coupled with a K-probe which enabled measurement up to 0.3 m subsurface (Table S2). Surface temperatures at thermally inactive sites reflected the weather and ambient air temperatures.

### Identification of efflorescence compositions

Efflorescences blooming at fissures were identified using SEM–EDS and XRD. The morphologies of samples on carbon tape were examined using a Philips XL 30 ESEM/TMP scanning electron microscope coupled to an energy-dispersive spectrometer (EDS; EDAX type Sapphire) at the Faculty of Earth Sciences, University of Silesia. Analytical conditions were: accelerating voltage 15 kV, working distance ca 10 mm, and counting time 40 s. In addition, powdered samples were examined using a Bruker AXS D8 ADVANCE diffractometer in the X-Ray Diffraction Laboratory, Institute of Geological Sciences, Polish Academy of Sciences and an X-ray Philips PW 3710 diffractometer at the Faculty of Earth Sciences, University of Silesia. Efflorescence phase compositions are given in Table S2.

### Gas chromatography

To assess the variability of gas compositions, several dominating compounds were selected based on previous research (Fabiańska et al. 2013). Molecular compositions of self-heating gases (CH_4_, C_2_H_6_, C_3_H_8_, *i*C_4_H_10_, *n*C_4_H_10_, C_5_H_12_, C_6_H_14_, C_7_H_16_, unsaturated hydrocarbons, CO_2_, O_2_, H_2_, N_2_) were determined on an Agilent 7890A gas chromatograph equipped with a set of columns, and flame ionization (FID) and thermal conductivity (TCD) detectors. This GC is configured to do an extended natural gas analysis up to C_14_. The analyzer is a three-valve system using three 1/8-inch packed columns (3 ft Hayesep Q 80/100 mesh, 6 ft Hayesep Q 80/100 mesh, and 10 ft molecular sieve 13 × 45/60 mesh) and a GS-Alumina capillary column (50 m × 0.53 mm). The system consists of two independent channels. The channel using the FID for the detailed hydrocarbon analysis is a simple gas sampling valve injecting the sample into the GS-Alumina column. The second channel using packed columns is for determination of methane, ethane, and non-hydrocarbon gases. The GC oven was programmed as follows: initial T of 60 °C held for 1 min., then to 90 °C at 10 °C/min., then to 190 °C at 20 °C/min., and finally held for 5 min. The front detector (TCD) was operated at 150 °C, and the back detector (FID) at 250 °C. Helium was used as a carrier gas flowing through the TCD channel at cm^3^ min^−1^ and through the FID channel at cm^3^ min^−1^.

## Results and discussion

### General composition of gases

Almost all samples contained gaseous products resulting from the thermal destruction of coal waste organic matter mixed with atmospheric oxygen and nitrogen. Total concentrations of gases show high variability related to the sampling site and self-heating stage. Obviously, the highest absolute emissions occur in very active sites such as at the Anna, Wełnowiec, and Rymer Cones, whereas emissions from dumps showing low thermal activity, or none as the Przygórze, are hundreds of times lower (Table S3). In terms of the temperatures measured at sampling sites (Table S2), and of the self-heating stage, the gas samples may be grouped into samples from (1) from sites with no thermal activity, i.e., with no known fire history or where thermal activity has ceased, (2) sites with self-heating ongoing; these subdivide into gases (a) from the initial stage of self-heating, (b) emitted during intense heating, and (c) from sites of waning activity.

Apart from O_2_ and N_2_, two further main components are CH_4_ and CO_2_, both toxic and regarded as the main greenhouse gases (Kim 2007; EPA 2005). Absolute gas concentration values (Table S3) give information about emission scale, whereas relative percentage compositions show correlations between components. Compared to CH_4_, all other hydrocarbons appear in much lower amounts (Table S3). Unsaturated hydrocarbons were typically present in lower amounts than were their saturated analogues. To compare the highly variable gas distributions, the following relative percentage concentrations were calculated: (1) The relative percentage compositions of gases present together with atmospheric N_2_ and O_2_ (Table 1a, b), (2) relative percentage compositions of organic compounds, including CH_4_ (Table 2), (3) relative percentage compositions of heavier hydrocarbons, excluding CH_4_. As there are significant differences in emitted gas compositions between the Upper (US) and Lower Silesia (LS) basins, both are treated separately below.

**Table 1:** Relative percentage contents of gases in coal waste dump emissions: (a) hydrocarbons, (b) atmospheric gases (vol.%)

Sample code	Methane	Ethane	Ethylene	Propane	Propylene	*i*-Butane	*n*-Butane	Acetylene	*trans*-2-Butene	1-Butene	*cis*-2-Butene	*i*-Pentane	*n*-Pentane	*n*-Hexane
1a														
*Upper Silesia—the Wełnowiec coal waste dump*														
W1	0.000641	0.000139	0.000457	0.000051	–	0.000034	–	0.000113	–	–	0.001574	0.000068	–	–
W2a	0.064420	0.005277	0.001121	0.000617	0.000187	0.000063	0.000185	–	–	–	0.001062	0.000139	–	–
W2b	0.084270	0.007090	0.001596	0.001022	0.000253	0.000103	0.000444	–	–	–	0.001408	0.000130	0.000152	–
W3a	1.255880	0.022431	0.002208	0.000928	0.000327	0.000062	0.000164	–	–	–	0.001637	0.000092	–	–
W3b	0.234254	0.036257	0.002236	0.004768	0.000046	0.000305	0.001257	–	–	–	0.001106	0.000230	0.000192	–
W4a	0.074600	0.002030	0.000799	0.000227	0.000181	–	0.000114	–	–	–	0.001416	–	–	–
W4b	0.339053	0.000761	0.000541	–	–	–	–	–	–	–	–	0.000586	0.000177	–
W5	8.294222	0.045178	0.003540	0.004944	0.000829	0.000667	0.001387	0.001074	0.000220	0.000171	0.001876	0.000375	0.000278	0.000070
W6a	6.560138	0.003211	0.001517	0.000371	0.000114	0.000046	0.000112	–	–	–	0.000393	0.000082	–	–
W6b	7.849580	0.004710	0.001418	0.000573	0.000282	0.000060	0.000177	–	–	–	0.000937	0.000142	–	–
W7	0.000919	–	0.000349	–	–	–	–	–	–	–	–	0.000507	0.000179	0.000075
W8	0.000268	–	0.000093	–	–	–	–	–	–	–	–	0.000516	0.000151	0.000035
**Aver. active W2–W6**	**2.75071**	**0.01411**	**0.00166**	**0.00149**	**0.00025**	**0.00015**	**0.00043**	**0.00012**	**0.00002**	**0.00002**	**0.00123**	**0.00022**	**0.00009**	**0.00001**
**Aver. inactive W1, W7, W8**	**0.00061**	**–**	**0.00030**	**–**	**–**	**–**	**–**	**–**	**–**	**–**	**–**	**0.00036**	**0.00011**	**0.00004**
*Upper Silesia—the Rymer Cones coal waste dump*														
R1a	0.617462	0.048014	0.000903	0.013604	0.001860	0.001541	0.003135	–	0.000319	0.000319	0.000691	0.000850	0.001382	0.001010
R1b	0.633700	0.024700	0.003300	0.006000	0.003200	0.000700	0.001100	–	–	0.000200	–	0.000200	0.000200	0.000100
R2	0.012441	0.002584	0.000000	0.002984	0.000533	0.000533	0.001012	–	0.000107	0.000160	0.001279	0.000426	0.000906	0.000480
R3	0.089900	0.003700	0.000513	0.000900	0.000500	0.000100	0.000200	–	–	0.000029	–	0.000040	0.000036	0.000018
R4a	1.688008	0.159600	0.006800	0.050650	0.012250	0.005550	0.012100	–	0.002000	0.001800	0.000850	0.003150	0.005450	0.003350
R4b	0.100500	0.006000	0.000500	0.001500	0.000551	0.000168	0.000300	–	–	0.000044	–	0.000100	0.000100	0.000016
R5a	1.162088	0.105668	0.002459	0.031708	0.004761	0.003453	0.007273	–	0.000785	0.000733	0.000523	0.001884	0.003192	0.002093
R5b	0.023900	0.001800	0.000200	0.000500	0.000200	0.000100	0.000100	–	–	0.000100	–	0.000032	0.000037	0.000026
R6a	1.455991	0.104682	0.002494	0.029560	0.004780	0.003429	0.006494	–	0.000935	0.000727	0.000520	0.001766	0.002805	0.001818
R6b	0.006700	0.000700	0.000200	0.000100	0.000100	–	–	–	–	0.000120	–	0.000014	0.000010	0.000014
**Aver. all active**	**0.579069**	**0.045745**	**0.001737**	**0.013751**	**0.002874**	**0.001557**	**0.003171**	**–**	**0.000415**	**0.000423**	**0.000386**	**0.000846**	**0.001412**	**0.000893**
*Upper Silesia—the Anna coal waste dump*														
A1	0.159249	0.001136	0.000423	0.001716	0.000088	–	–	–	–	0.000016	0.000018	–	–	–
A2	0.075632	0.000493	0.000189	0.001652	0.000032	–	–	–	–	–	0.000008	–	–	–
A3	0.010689	0.000052	0.000006	0.001488	–	–	–	0.001933	–	–	0.000010	–	–	0.000715
A4	0.426349	0.005325	0.000072	0.003347	0.000038	0.000031	**–**	0.001868	0.000048	0.000047	0.000061	0.000070	0.000041	0.001725
A5	0.001817	0.000009	–	0.001904	–	–	–	–	–	–	–	–	–	–
**Aver. all active**	**0.134747**	**0.001403**	**0.000138**	**0.002021**	**0.000032**	**0.000006**	**–**	**0.000760**	**0.000010**	**0.000013**	**0.000019**	**0.000014**	**0.000008**	**0.000488**
*Upper Silesia—the Czerwionka-Leszczyny coal waste dump*														
CzL1	0.337707	0.000057	0.000013	0.001689	–	–	–	0.002420	**–**	**–**	–	–	–	0.001305
CzL2	0.026756	0.003823	0.000063	0.001829	0.000017	0.000075	**–**	**–**	**–**	**–**	**–**	0.000017	0.000004	–
CzL3	0.014712	0.000165	0.000007	0.001644	–	–	–	–	**–**	**–**	–	–	–	–
CzL4	0.021403	0.000260	0.000027	0.001667	0.000008	0.000020	–	–	–	–	–	–	–	–
CzL5	0.014073	0.000078	–	0.000816	–	–	–	–	**–**	**–**	–	–	–	–
**Aver. all active**	**0.082930**	**0.000877**	**0.000022**	**0.001529**	**0.000005**	**0.000019**	**0.000000**	**0.000484**	**0.000000**	**0.000000**	**0.000000**	**0.000003**	**0.000001**	**0.000261**
*Lower Silesia—the Słupiec coal waste dump*														
S1a	0.197011	0.000941	–	0.000375	0.000105	0.001267	0.001252	0.000884	0.002364	0.004547	0.002360	0.000724	0.000380	0.000029
S1b	0.011845	0.000190	–	0.000048	–	0.000162	0.000235	–	0.000241	0.000288	0.000245	0.000191	0.000168	0.000025
S1c	0.259840	0.000123	–	0.000023	–	0.000124	0.000193	–	0.000193	0.000307	0.000214	0.000190	0.000109	0.000000
S1d	0.311703	0.023167	0.002863	0.003194	0.001252	0.000375	0.001213	–	0.000345	0.000440	0.000279	0.000273	0.000349	0.000000
S1e	0.432542	0.014020	0.000701	0.001901	0.000470	0.000243	0.000886	–	0.000197	0.000181	0.000258	0.000206	0.000277	0.000079
S1f	0.265099	0.008927	0.000320	0.001121	0.000376	0.000190	0.000500	–	0.000117	–	0.000168	0.000155	0.000151	–
S1g	0.151282	0.014245	0.001366	0.002033	0.000768	0.000291	0.000870	–	0.000245	0.000148	0.000355	0.000213	0.000272	–
S2a	0.585917	0.015567	0.001970	0.002121	0.000971	0.000416	0.000114	0.000414	0.000438	0.000155	0.000423	0.000365	0.000391	0.000101
S2b	0.877982	0.017739	0.001794	0.001794	0.001127	0.000428	0.001260	0.000077	0.000522	0.000178	0.000530	0.000358	0.000414	0.000109
S3	0.558831	0.019393	0.001613	0.002803	0.001100	0.000355	0.001355	–	0.000455	0.000245	0.000567	0.000286	0.000420	0.000090
S4a	0.123534	0.007369	0.000066	0.002081	0.000139	0.000969	0.001544	–	–	–	0.000118	0.000813	0.000518	0.000187
S4b	0.232282	0.009787	0.000319	0.001660	0.000315	0.000340	0.000849	–	0.000113	–	0.000213	0.000233	0.000231	0.000074
S5a	0.454337	0.001482	–	0.001299	0.000072	0.000869	0.001581	–	–	–	0.000129	0.000812	0.000631	0.000202
S6	0.237397	–	–	0.000024	–	–	–	–	–	–	–	–	–	–
S7	0.500239	0.000243	–	0.000065	0.000031	0.000042	–	–	–	–	0.000352	–	–	–
S8a	0.102990	0.009811	0.000168	0.001424	0.000365	0.000193	0.000544	–	0.000145		0.000211	0.000126	0.000163	–
S8b	0.186441	0.009785	0.000212	0.001513	0.000420	0.000204	0.000700	–	0.000248	0.000081	0.000277	0.000159	0.000225	0.000073
S9	0.144998	0.008616	0.000764	0.001243	0.000230	0.000222	0.000580	–	–	–	0.001073	0.000242	0.000244	0.000086
S10a	0.012136	0.000323	–	0.000033	–	–	–	–	–	–	–	–	–	–
S10b	0.001787	0.001680	0.001248	–	–	–	–	0.001424	–	–	–	–	–	–
S11	0.000335	0.000082	0.000199	–	–	–	–	–	–	–	–	0.000410	0.000192	0.000045
**Aver. active**	**0.27001**	**0.00817**	**0.00067**	**0.00124**	**0.00039**	**0.00034**	**0.00069**	**0.00014**	**0.00028**	**0.00036**	**0.00039**	**0.00027**	**0.00025**	**0.00006**
**S1–S10 inactive S11**	**0.000335**	**0.000082**	**0.000199**	–	–	–	–	–	–	–	–	**0.000410**	**0.000192**	**0.000045**
*Lower Silesia—the Nowa Ruda and Przygórze coal waste dumps*														
N1	0.007552	0.000227	0.001604	0.000438	0.000054	0.003155	0.001838	–	0.003529	0.006512	0.003900	0.003182	0.001506	0.000093
N2	0.016100	0.000045	0.001904	0.000012	–	0.000016	0.000022	–	–	–	0.000459	0.000368	0.000146	0.000116
N3	0.025587	0.000121	0.001666	0.000012	–	0.000009	0.000021	–	–	–	0.000734	0.000353	0.000132	0.000100
P1	0.007052	–	0.001928	0.000010	–	0.000012	0.000023	–	–	–	0.000599	0.000370	0.000136	0.000115
**Aver. active N2 and N3**	**0.02084**	**0.00008**	**0.00179**	**0.00001**	**–**	**0.00001**	**0.00002**	**–**	**–**	**–**	**0.00060**	**0.00036**	**0.00014**	**0.00011**
**Aver. inactive N1, P1**	**0.00730**	**0.00011**	**0.00177**	**0.00022**	**0.00003**	**0.00158**	**0.00093**	**0.00000**	**0.00176**	**0.00326**	**0.00225**	**0.00178**	**0.00082**	**0.00010**
**US aver. active**	**0.88686**	**0.01553**	**0.00089**	**0.00470**	**0.00079**	**0.00043**	**0.00120**	**0.00045**	**0.00011**	**0.00011**	**0.00041**	**0.00027**	**0.00038**	**0.00041**
**US aver. inactive**	**0.00061**	**–**	**0.00030**	**–**	**–**	**–**	**–**	**–**	**–**	**–**	**–**	**0.00036**	**0.00011**	**0.00004**
**LS aver. active**	**0.14543**	**0.00413**	**0.00123**	**0.00063**	**0.00039**	**0.00017**	**0.00035**	**0.00014**	**0.00028**	**0.00036**	**0.00049**	**0.00031**	**0.00019**	**0.00008**
**LS aver. inactive**	**0.00382**	**0.00010**	**0.00098**	**0.00022**	**0.00003**	**0.00158**	**0.00093**	**0.00000**	**0.00176**	**0.00326**	**0.00225**	**0.00109**	**0.00051**	**0.00007**

Sample code	Hydrogen	Carbon dioxide	Oxygen	Nitrogen
1b				
*Upper Silesia—the Wełnowiec coal waste dump*				
W1	–	3.254901	14.203726	82.538294
W2a	0.021811	2.633048	14.800306	82.471765
W2b	0.041868	8.279071	5.365313	86.217279
W3a	2.475863	6.860296	6.922538	82.457574
W3b	0.031623	5.262211	9.010453	85.415062
W4a	–	2.277235	17.880625	79.762774
W4b	0.875826	6.263069	6.072594	86.447393
W5	0.069703	5.714674	6.598215	79.262576
W6a	–	5.593478	7.086179	80.754360
W6b	0.120757	5.567367	6.760775	79.693222
W7	–	–	21.058753	78.939218
W8	–	–	21.150359	78.848579
**Aver. active W2–W6**	**0.519636**	**5.383383**	**8.944111**	**82.498000**
**Aver. inactive W1, W7, W8**	**–**	**1.084967**	**18.804279**	**80.108697**
*Upper Silesia—the Rymer Cones coal waste dump*				
R1a	0.189931	7.037494	12.551819	77.529665
R1b	0.156100	10.027900	9.308900	79.833000
R2	–	0.489473	20.272945	77.201725
R3	0.066700	1.358800	18.881400	79.597000
R4a	0.272033	14.837700	2.734802	74.108405
R4b	0.049300	1.114300	19.40300	79.323900
R5a	0.186012	9.058528	9.650628	76.604483
R5b	0.020400	0.789100	19.880500	79.282900
R6a	0.507773	14.638253	2.601312	77.121080
R6b	0.014000	0.599700	20.046300	79.331800
**Aver. all active**	**0.159460**	**6.056626**	**13.313992**	**77.909938**
*Upper Silesia—the Anna coal waste dump*				
A1	0.123980	6.563265	8.177945	79.300150
A2	0.008085	0.897500	18.349540	74.011555
A3	0.012689	3.826520	14.840005	77.631035
A4	0.247847	4.747250	13.809425	79.480005
A5	0.010801	4.178760	14.391250	81.410150
**Aver. all active**	**0.080680**	**4.042659**	**13.913633**	**78.366579**
*Upper Silesia—the Czerwionka-Leszczyny coal waste dump*				
CzL1	–	0.052637	19.765595	73.849975
CzL2	0.005350	1.201535	17.630995	75.488860
CzL3	–	0.183748	19.636245	74.310320
CzL4	0.001680	0.504425	19.135430	74.659050
CzL5	0.000286	0.414928	19.280920	74.552090
**Aver. all active**	**0.001463**	**0.471455**	**19.089837**	**74.572059**
*Lower Silesia—the Słupiec coal waste dump*				
S1a	–	3.792767	15.70888	80.286114
S1b	–	4.460009	14.41442	81.111933
S1c	–	6.410055	12.315451	81.013287
S1d	0.164974	9.296371	5.911334	84.281870
S1e	–	14.090058	2.594536	82.863443
S1f	–	12.166156	2.936752	84.619969
S1g	0.169160	12.099068	3.214514	84.345167
S2a	0.009455	12.714460	3.346275	83.320454
S2b	–	13.263222	2.782431	83.050037
S3	0.062048	13.452881	2.476170	83.421390
S4a	–	10.019199	4.387025	85.456438
S4b	0.192721	11.951113	3.630384	83.979369
S5a	–	10.414962	5.625016	83.498606
S6	–	2.186160	17.81778	79.758640
S7	–	11.583891	3.904533	84.010609
S8a	0.015602	9.4614199	4.334994	86.071854
S8b	0.023925	11.281991	2.962421	85.531340
S9	0.014090	9.445939	4.183020	86.198650
S10a	–	9.131870	9.525450	81.330189
S10b	–	0.217898	21.21460	78.561561
S11	–	–	21.20241	78.796332
**Aver. active**	**0.031046**	**9.274462**	**7.269247**	**83.140478**
**S1**–**S10 inactive S11**	–	–	**21.202411**	**78.796330**
*Lower Silesia—the Nowa Ruda and Przygórze coal waste dumps*				
N1	–	–	20.598570	78.467280
N2	–	1.646410	18.061007	78.688582
N3	0.013953	1.998198	17.928787	79.315631
P1	–	0.302372	20.369375	78.398474
**Aver. active N2 and N3**	**0.006976**	**1.822304**	**17.994897**	**79.002105**
**Aver. inactive N1, P1**	**–**	**0.151186**	**20.483972**	**78.432875**
**US aver. active**	**0.158139**	**3.973144**	**13.870198**	**78.357500**
**US aver. inactive**	**–**	**1.084967**	**18.804279**	**80.108697**
**LS aver. active**	**0.019011**	**5.548383**	**12.632072**	**81.071292**
**LS aver. inactive**	**–**	**0.075593**	**20.843191**	**78.614603**

Averages are shown in bold

“–” Compounds were not found

**Table 2:** Relative percentage contents of gaseous hydrocarbons in coal waste dump emissions

Sample code	Methane	Ethane	Ethylene	Propane	Propylene	*i*-Butane	*n*-Butane	Acetylene	*trans*-2-Butene	1-Butene	*cis*-2-Butene	*i*-Pentane	*n*-Pentane	*n*-Hexane
*Upper Silesia—the Wełnowiec coal waste dump*														
W1	20.84	4.53	14.86	1.67	0.00	1.10	0.00	3.67	–	–	51.13	2.21	–	–
W2a	88.16	7.22	1.53	0.85	0.26	0.09	0.25	–	–	–	1.45	0.19	–	–
W2b	87.35	7.35	1.65	1.06	0.26	0.11	0.46	–	–	–	1.46	0.13	0.16	–
W3a	97.83	1.75	0.17	0.07	0.03	–	0.01	–	–	–	0.13	0.01	–	–
W3b	83.47	12.92	0.80	1.70	0.02	0.11	0.45	–	–	–	0.39	0.08	0.07	–
W4a	93.99	2.56	1.01	0.29	0.23	–	0.14	–	–	–	1.78	–	–	–
W4b	99.39	0.22	0.16	–	–	–	–	–	–	–	–	0.17	0.05	–
W5	99.27	0.54	0.04	0.06	0.01	0.01	0.02	0.01	–	–	0.02	–	–	–
W6a	99.91	0.05	0.02	0.01	–	–	–	–	–	–	0.01	–	–	–
W6b	99.89	0.06	0.02	0.01	–	–	–	–	–	–	0.01	–	–	–
W7	45.28	–	17.22	–	–	–	–	–	–	–	–	24.99	8.82	3.69
W8	25.20	–	8.77	–	–	–	–	–	–	–	–	48.56	14.21	3.25
**Aver. active W2**–**W6**	**94.36**	**3.63**	**0.60**	**0.45**	**0.09**	**0.04**	**0.15**	**0.01**	–	–	**0.58**	**0.06**	**0.03**	–
**Aver. inactive W1, W7, W8**	**30.44**	**1.51**	**13.62**	**0.56**	**0.00**	**0.37**	**0.00**	**1.22**	**0.00**	**0.00**	**17.04**	**25.25**	**7.68**	**2.31**
*Upper Silesia—the Rymer Cones coal waste dump*														
R1a	89.35	6.95	0.13	1.97	0.27	0.22	0.45	–	0.05	0.05	0.10	0.12	0.20	0.15
R1b	94.12	3.67	0.49	0.89	0.48	0.10	0.16	–	–	0.03	–	0.03	0.03	0.01
R2	53.07	11.02	0.00	12.73	2.27	2.27	4.32	–	0.45	0.68	5.45	1.82	3.86	2.05
R3	93.71	3.86	0.53	0.94	0.52	0.10	0.21	–	–	0.03	–	0.04	0.04	–
R4a	86.50	8.18	0.35	2.60	0.63	0.28	0.62	–	0.10	0.09	0.04	0.16	0.28	0.17
R4b	91.55	5.47	0.46	1.37	0.50	0.15	0.27	–	–	0.04	–	0.09	0.09	–
R5a	87.60	7.97	0.19	2.39	0.36	0.26	0.55	–	0.06	0.06	0.04	0.14	0.24	0.16
R5b	88.54	6.67	0.74	1.85	0.74	0.37	0.37	–	–	0.37	–	0.12	0.14	–
R6a	90.10	6.48	0.15	1.83	0.30	0.21	0.40	–	0.06	0.05	0.03	0.11	0.17	0.11
R6b	84.19	8.80	2.51	1.26	1.26	–	–	–	–	–	–	0.18	0.13	0.18
**Aver. all active**	**85.87**	**6.91**	**0.56**	**2.78**	**0.73**	**0.40**	**0.74**	**0.00**	**0.07**	**0.29**	**0.57**	**0.28**	**0.52**	**0.28**
*Upper Silesia—the Anna coal waste dump*														
A1	97.91	0.70	0.26	1.06	0.05	–	–	–	–	0.01	0.01	–	–	–
A2	96.96	0.63	0.24	2.12	0.04	–	–	–	–	–	0.01	–	–	–
A3	71.77	0.35	0.04	9.99	–	–	–	12.98	–	–	0.06	–	–	4.80
A4	97.11	1.21	0.02	0.76	0.01	0.01	–	0.43	0.01	0.01	0.01	0.02	0.01	0.39
A5	48.72	0.24	–	51.04	–	–	–	–	–	–	–	–	–	–
**Aver. all active**	**82.49**	**0.63**	**0.11**	**12.99**	**0.02**	–	–	**2.68**	–	**0.00**	**0.02**	–	–	**1.04**
*Upper Silesia—the Czerwionka-Leszczyny coal waste dump*														
CL1	98.40	0.02	0.00	0.49	–	–	–	0.71	–	–	–	–	–	0.38
CL2	82.11	11.73	0.19	5.61	0.05	0.23	–	–	–	–	–	0.05	0.01	–
CL3	89.01	1.00	0.04	9.95	–	–	–	–	–	–	–	–	–	–
CL4	91.52	1.11	0.12	7.13	0.04	0.08	–	–	–	–	–	–	–	–
CL5	94.02	0.52	–	5.45	–	–	–	–	–	–	–	–	–	–
**Aver. all active**	**91.01**	**2.88**	**0.07**	**5.73**	**0.02**	**0.06**	–	**0.14**	–	–	–	**0.01**	**0.00**	**0.08**
*Lower Silesia—the Słupiec coal waste dump*														
S1a	92.82	0.44	–	0.18	0.05	0.60	0.59	0.42	1.11	2.14	1.11	0.34	0.18	0.01
S1b	86.85	1.39	–	0.35	–	1.19	1.73	–	1.77	2.11	1.79	1.40	1.23	0.18
S1c	86.85	1.39	–	0.35	–	1.19	1.73	–	1.77	2.11	1.79	1.40	1.23	0.18
S1d	90.23	6.71	0.83	0.92	0.36	0.11	0.35	–	0.10	0.13	0.08	0.08	0.10	–
S1e	95.70	3.10	0.16	0.42	0.10	0.05	0.20	–	0.04	0.04	0.06	0.05	0.06	0.02
S1f	95.66	3.22	0.12	0.40	0.14	0.07	0.18	–	0.04	–	0.06	0.06	0.05	–
S1g	87.91	8.28	0.79	1.18	0.45	0.17	0.51	–	0.14	0.09	0.21	0.12	0.16	–
S2a	96.15	2.55	0.32	0.35	0.16	0.07	0.02	0.07	0.07	0.03	0.07	0.06	0.06	0.02
S2b	97.09	1.96	0.20	0.20	0.12	0.05	0.14	0.01	0.06	0.02	0.06	0.04	0.05	0.01
S3	95.12	3.30	0.27	0.48	0.19	0.06	0.23	–	0.08	0.04	0.10	0.05	0.07	0.02
S4a	89.95	5.37	0.05	1.52	0.10	0.71	1.12	–	–	–	0.09	0.59	0.38	0.14
S4b	94.26	3.97	0.13	0.67	0.13	0.14	0.34	–	0.05	–	0.09	0.09	0.09	0.03
S5a	98.47	0.32	–	0.28	0.02	0.19	0.34	–	–	–	0.03	0.18	0.14	0.04
S6	99.99	–	–	0.01	–	–	–	–	–	–	–	–	–	–
S7	99.85	0.05	–	0.01	0.01	0.01	–	–	–	–	0.07	–	–	–
S8a	88.68	8.45	0.14	1.23	0.31	0.17	0.47	–	0.13	–	0.18	0.11	0.14	–
S8b	93.06	4.88	0.11	0.76	0.21	0.10	0.35	–	0.12	0.04	0.14	0.08	0.11	0.04
S9	91.60	5.44	0.48	0.79	0.15	0.14	0.37	–	–	–	0.68	0.15	0.15	0.05
S10a	97.15	2.58	–	0.26	–	–	–	–	–	–	–	–	–	–
S10b	29.11	27.37	20.33	–	–	–	–	23.19	–	–	–	–	–	–
S11	26.53	6.46	15.77	–	–	–	–	–	–	–	–	32.51	15.18	3.55
**Aver. active S1**–**S10**	**90.33**	**4.54**	**1.20**	**0.52**	**0.13**	**0.25**	**0.43**	**1.18**	**0.27**	**0.34**	**0.33**	**0.24**	**0.21**	**0.04**
**inactive S11**	**26.53**	**6.46**	**15.77**	–	–	–	–	–	–	–	–	**32.51**	**15.18**	**3.55**
*Lower Silesia—the Nowa Ruda and Przygórze coal waste dumps*														
N1	22.48	0.68	4.78	1.31	0.16	9.39	5.47	–	10.50	19.39	11.61	9.47	4.48	0.28
N2	83.67	0.23	9.89	0.06	–	0.08	0.12	–	–	–	2.39	1.91	0.76	0.60
N3	88.86	0.42	5.79	0.04	–	0.03	0.07	–	–	–	2.55	1.23	0.46	0.35
P1	68.37	–	18.69	0.10	–	0.11	0.22	–	–	–	5.81	3.59	1.32	1.12
**Aver. active N2 and N3**	**86.26**	**0.33**	**7.84**	**0.05**	–	**0.055**	**0.10**	–	–	–	**2.47**	**1.57**	**0.61**	**0.47**
**Aver. inactive N1, P1**	**45.42**	**0.34**	**11.73**	**0.71**	**0.08**	**4.75**	**2.85**	–	**5.25**	**9.69**	**8.71**	**6.53**	**2.90**	**0.70**
**US aver. active**	**88.44**	**3.51**	**0.33**	**5.49**	**0.22**	**0.13**	**0.29**	**0.71**	**0.04**	**0.15**	**0.39**	**0.09**	**0.14**	**0.47**
**US aver. inactive**	**30.44**	**1.51**	**13.62**	**0.56**	–	**0.37**	–	**1.22**	–	–	**17.04**	**25.25**	**7.68**	**2.31**
**LS aver. active**	**88.30**	**2.43**	**4.52**	**0.28**	**0.13**	**0.15**	**0.26**	**1.18**	**0.27**	**0.34**	**1.40**	**0.91**	**0.41**	**0.26**
**LS aver. inactive**	**35.98**	**3.40**	**13.75**	**0.71**	**0.08**	**4.75**	**2.85**	–	**5.25**	**9.70**	**8.71**	**19.52**	**9.04**	**2.13**

Averages are shown in bold

“–” Compounds were not found

The typical gas at a site with no thermal activity is dominated by atmospheric O_2_ and N_2_ (Tables 1 and S3). Average percentage contents for the US and LS basins are: N_2_ = 78.6 and 80.1% vol., respectively, and O_2_ = 18.8 and 20.8% vol., respectively. Carbon dioxide contents are elevated compared to average atmosphere (0.035% vol.), namely 1.085 (US) and 0.151 (LS) % vol. However, at some inactive sites CO_2_ was absent. The atmospheric gases are accompanied by small amounts of organic compounds, among which, CH_4_ (0.0061 and 0.0040% vol.) and ethylene (0.0030 and 0.0011% vol.) predominate. Heavier aliphatic hydrocarbons from *cis*-2-butene to *n*-hexane occur in much lower amounts (0.0001–0.0005% vol.).

Gases from ongoing self-heating sites show a significant decrease in O_2_ content, being < 5.5 times lower than in the atmosphere. Apart from the major atmospheric gases, CO_2_ is the predominating component, averaging 3.7350 (US) and 5.2447 (LS) % vol. The organic gases also include CH_4_ (1.3233 (US) and 0.1432 (LS) % vol.), saturated aliphatic hydrocarbons including ethane, propane, *n*-butane, *n*-pentane, *n*-hexane, *n*-heptane, *iso*-butane, and *iso*-pentane, together with unsaturated aliphatic hydrocarbons including ethylene, acetylene, propylene, and *trans*- and *cis*-2-butene. Typically, concentrations decrease with increasing molecular weight but, in some LS gases (S1a, S1d, S2b, S3, and S4a), elevated contents of propane and *n*-butane were noted. In sample A1, the relative content of propane exceeds that of CH_4_ (Table S3). Thermal activity also results in elevated H_2_ contents, i.e., 0.2125 (US) and 0.0186 (LS) % vol. (Table 1b). These values greatly exceed average atmospheric H_2_ concentrations (0.0000055% vol.). The unsaturated hydrocarbons and H_2_ are pyrolytical products of self-heating; they are common in refinery and coal pyrolysis gases (Saavedra et al. 2013; Speight 2014).

### Gas compositions, emission levels, and their potential significance

In the dump emissions, the classes of gases distinguished include (1) main air components, (2) oxygenated compounds (CO_2_), (3) reducing gases (CH_4_ and H_2_), (4) saturated aliphatic hydrocarbons in the range C_2_–C_7_, and (5) unsaturated aliphatic hydrocarbons in the range C_2_–C_4_.

### Carbon dioxide

Apart from oxygen and nitrogen, the predominating component of all gases from sites with ongoing thermal activity is CO_2_ present in amounts < several relative percent (vol.). Apart from its significance as a greenhouse gas, CO_2_ is also toxic. The normal CO_2_ concentration outdoors is ca 300–350 ppm or 0.54–0.63 g/m^3^ (Killops and Killops 2005). The level still comfortable indoors is 600–800 ppm (1.08–1.44 g/m^3^). The highest CO_2_ concentration registered at thermally active sites was 291.5211 g/m^3^ or 161,666 ppm. This is > 500 times the normal atmospheric level, and > 1.5 times the level (100 000 ppm) that leads to loss of consciousness and, ultimately, death (Brake and Bates 1999). This will happen even when O_2_ is at the normal atmospheric level, not the case in coal waste dump gas characterized by a significant decrease in O_2_ (Tables 1 and S3).

Carbon dioxide emissions from sites presently inactive but active in the past are 21.3167 (US) and 4.4556 (LS) g/m^3^ (aver. 12.8862 g/m^3^; Table S3). Thus, CO_2_ emissions from inactive dumps at ca 7000 ppm are close to levels at which adverse health effects might be expected (10 000 ppm; ACGIH 1999; Pauluhn 2016). Moreover, CO_2_ is considered to aggravate the toxicity of CO when both are present in the same gas (Pauluhn 2016). This suggests that the use of apparently inactive coal waste dumps as recreation sites may involve harmful exposure levels.

**Fig. 3:**
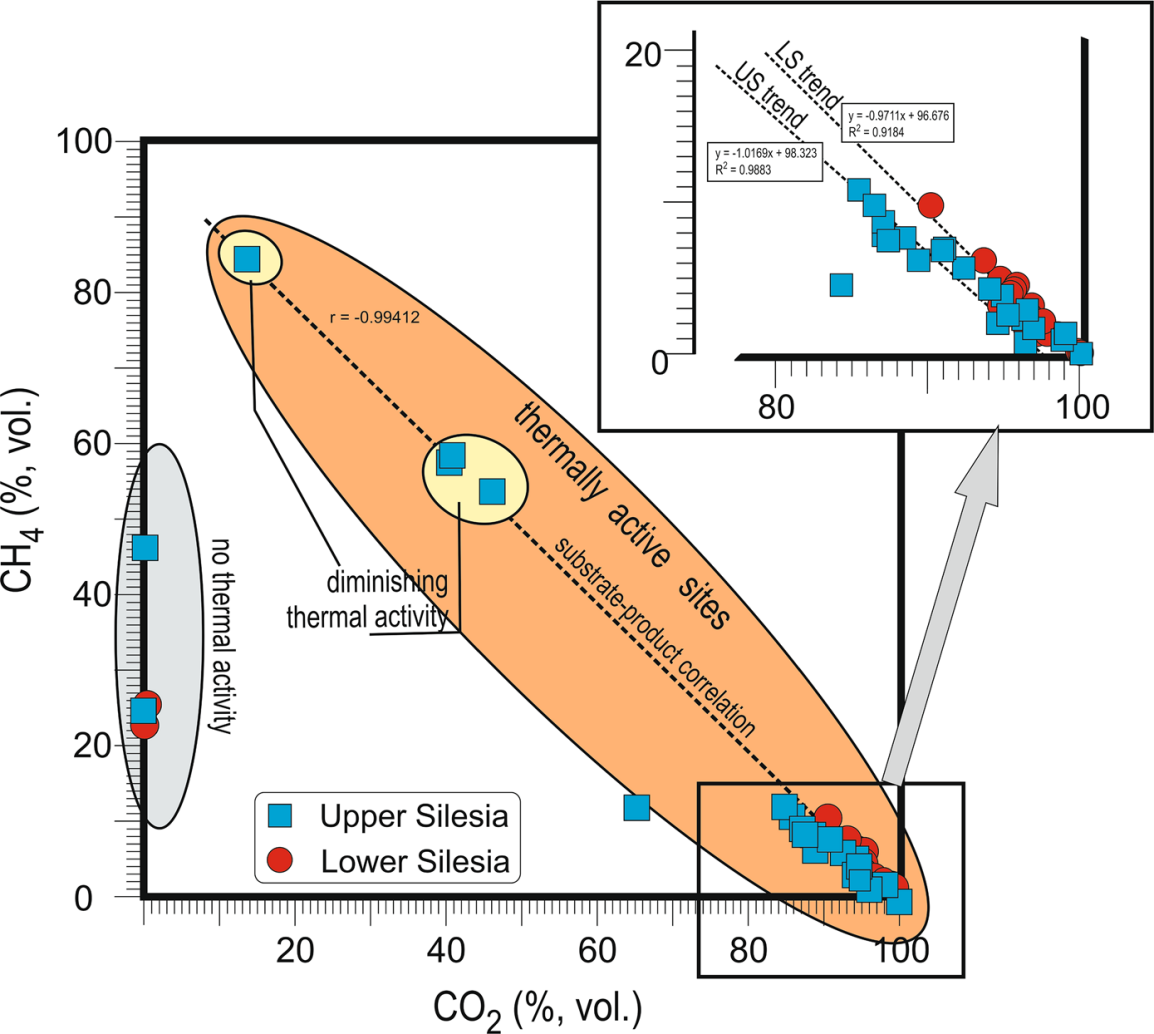
Correlation between CO_2_ and CH_4_ (rel.%) contents in coal waste dump gases

Carbon dioxide predominance in coal waste self-heating gases is common (Yan et al. 2003; Kim 2007; Carras et al. 2009; Hower et al. 2009; O’Keefe et al. 2010), with contents increasing significantly with increasing thermal activity. CO_2_ also shows inverse correlations with CH_4_ (below) when the emitted gas results from self-heating sites are recalculated to relative percentages, omitting N_2_ and O_2_ (Table 1; Fig. 3).

### Methane

Methane predominates among organic compounds, occurring in amounts > 80%, in some cases, < 99.91% rel. in sites of current thermal activity (Table 1a). It may be CH_4_ that was in coal pores as most US and LS coal mines are methane-rich (Kotarba 2001; Kędzior 2009), but is more likely related to organic matter cracking (Grossman et al. 1994; Davidi et al. 1995; Fabiańska et al. 2013). Methane is the only hydrocarbon occurring naturally in the atmosphere (1.6–1.8 ppm; Schneising et al. 2014; Dlugokencky 2016). This methane comes from the biosphere, e.g., wetlands, methanogenic microorganisms, and natural fires, and the geosphere, e.g., natural gas, volcanic eruptions, permafrost, or clathrates. Agriculture and the fossil fuel industry are responsible for the global increase from the pre-industrial value of 722 to 1800 ppb in 2016 (Schneising et al. 2014). Methane from shale gas production, measured over three shale regions in the USA, has increased the atmospheric level by ca 2.0 ppm, i.e., 0.0013 g/m^3^ (Peischl et al. 2015). The highest CH_4_ emissions from the Silesian dumps, recorded in Wełnowiec dump (W4b, W5, and W6), were a few tens g/m^3^. There are two possible explanations for these high CH_4_ levels, namely (i) the compound was released from still-decaying urban wastes originally dumped there, or (ii) that, during their combustion, the top cover of coal waste limited CH_4_ oxygenation. Conspicuously, lower CH_4_ emissions (from 0.1 to several g/m^3^) from other Silesian dumps are still 1000 times higher than atmospheric levels (Table S3).

Even at sites where fire was extinguished years ago, and ambient temperatures prevail, CH_4_ was recorded with emissions averaging 0.004363 (US) and 0.05163 (LS) g/m^3^ (6.7 and 78.9 ppm, respectively). These levels are 4–40 times atmospheric levels. However, they pale in comparison with thermally active sites where CH_4_ emissions average 6.350585 (US) and 1.031258 (LS) g/m^3^ (9704.5 and 1575.9 ppm, respectively). Carras et al. (2009), investigating emissions from Australian coal wastes without visible signs of combustion, found no methane but elevated CO_2_ concentrations. It is possible that, in Silesia, when self-heating has ended, CH_4_ continues to be emitted from pores, particularly if combustion conditions were reducing and oxygenation incomplete. Even more surprising is the CH_4_ presence at the reference site where heating never occurred (S11). Here, CH_4_ may be a biogenic product of microorganisms living on coal waste. It is usually considered that methanogens live in wet anaerobic conditions not seen in coal waste dumps (Tung et al. 2005). They are, however, found in extremely dry and oxic soils (Peters and Conrad 1995).

These data indicate that coal waste dumps, thermally active or not, should be considered a significant source of methane in industrial regions such as Silesia where ca 40 million tonnes of coal waste are produced annually (Korban 2011). The global significance of methane and CO_2_ fluxes from coal waste dumps may be underestimated.

### Carbon dioxide and methane relationship

Omitting N_2_ and O_2,_ as in Table 1, a clear relationship between the relative contents of CH_4_ and CO_2_ reveals an overall substrate–product relationship for thermally active sites (*r* = − 0.99; Fig. 3). This indicates that just after its release, CH_4_ oxidizes to CO_2_ within the dumps. The small difference in the correlation in the individual basins may relate to differences in the characteristics of the coal waste organic matter, e.g., rank, depositional environment or storage environment. Rank seems to be the more influential factor as two Upper Silesian dumps, Wełnowiec and Rymer Cones, correlate well despite their different shape, history, and size. The same substrate–product relationship exists between O_2_ and CO_2_ for both the US and LS basins (*r* = − 0.89 and − 0.98, respectively) and between values of oxygen decrease (OD) and CO_2_ relative contents (*r* = 0.87 and 0.93, respectively; Fig. 3).

Relative percentage contents of CO_2_ and CH_4_ seem to correlate with self-heating stage. Initial-stage sites, marked by organic efflorescences (W2 and W3), and sites with ongoing heating show no significant differences and CO_2_ production prevails (Table S2). However, where fire is beginning to wane (W5 and W6) CO_2_ relative contents decrease, whereas those of CH_4_ increase. No CO_2_ is expelled in thermally inactive sites.

### Saturated aliphatic hydrocarbons and unsaturated aliphatic hydrocarbons

Saturated aliphatic hydrocarbons occur in the range from ethane to heptane though, in most gases, C_6_ and C_7_ hydrocarbons are absent. Both normal and branched compounds occur. Apart from W1, W8, R1–4, S1a–c, S6, and S11, ethane predominates in the saturated gas fraction.

Unsaturated hydrocarbons comprise alkenes in the range C_2_–C_4_ and acetylene. Due to its relatively high reactivity, acetylene was found in only a few samples (W1, W5, A3, A4, CzL1, S1a, and S10b). Other compounds with triple bonds are absent. Among unsaturated hydrocarbons, ethylene dominates though typically comprising < 1.0% of total organic compounds. However, ethylene contents increase significantly in sites showing particularly elevated temperatures, e.g., to > 25% of all organic compounds in W3b (*t* = 690 °C at 50 cm). Contents of all other unsaturated hydrocarbons decrease with increasing carbon atom numbers in a molecule. Surprisingly, ethylene is also a significant component (< several %) of total organic components in gases at sites of waning thermal activity, e.g., W1, W7, W8, S1b, S11, and N1 with measured temperatures close to ambient. Possibly, as with methane, ethylene is degassed from pores even after self-heating ends or it is produced by bacteria growing on coal waste surfaces; many soil bacteria species, e.g., many chemolithotrophs, can produce ethylene (Nagahama et al. 1992).

Ethylene predominates over ethane in some once thermally active sites (W1, N1, N2, N3, and P1) where temperatures have waned to near ambient. Ethylene together with CO_2_ can markedly influence vegetation on coal waste dumps. Plants use CO_2_ to build tissues, and ethylene is a growth hormone accelerating flowering and fruit maturation (Johnson and Ecker 1998). The gigantism of the lush vegetation on self-heating dumps (Ciesielczuk et al. 2015) may thus be explained. Other unsaturated hydrocarbons present in much lower amounts include propylene and *cis*- and *trans*-2-butene. Though with toxicities less than those of CO_2_ and CH_4_, these are neurotoxins that, inhaled, cause dizziness, tachycardia, impaired coordination, and disorientation (Broussard 1999).

### Hydrogen and unsaturated hydrocarbons

Hydrogen was found only at thermally active sites, despite being a product of low-temperature oxidation of bituminous coals (Grossman et al. 1993; Czechowski et al. 2007). The inverse correlation of unsaturated hydrocarbons and free hydrogen indicates that double bonds are saturated in self-heating zones.

### Assessment of thermal activity level using gas ratios

To assess thermal activity in the coal waste dumps, and to compare its development in different dumps, the following ratios were calculated (Table 3).

**Table 3:** Component ratios characterizing variability of waste dump emissions

Sample code	N_2_/O_2_ (vol.)	OD^a^	CO_2_/CH_4_ (vol.)	Sat/Unsat.HC^b^	CH_4_/all HC^c^
*Upper Silesia—the Wełnowiec coal waste dump*					
W1	5.81	1.73	5074.30	0.14	0.26
W2a	5.57	1.66	40.87	2.65	7.45
W2b	16.07	4.80	98.24	2.74	6.91
W3a	11.91	3.56	5.46	5.68	45.10
W3b	9.48	2.83	22.46	12.69	5.05
W4a	4.46	1.33	30.53	0.99	15.65
W4b	14.24	4.25	18.47	2.82	164.27
W5	12.01	3.59	0.69	6.86	136.85
W6a	11.40	3.40	0.85	1.89	1122.26
W6b	11.79	3.52	0.71	2.15	945.73
W7	3.75	1.12	0.00	2.18	0.83
W8	3.73	1.11	0.00	7.53	0.34
**Aver. active W2**–**W6**	**10.77**	**3.22**	**24.25**	**4.27**	**272.14**
**Aver. inactive W1, W7, W8**	**4.43**	**1.32**	**1691.43**	**3.28**	**0.48**
*Upper Silesia—the Rymer Cones coal waste dump*					
R1a	6.18	1.84	11.40	156.35	23.38
R1b	8.58	2.56	15.82	4.91	16.00
R2	3.81	1.14	39.34	10.28	1.13
R3	4.22	1.26	15.11	4.79	14.89
R4a	27.10	8.09	8.79	81.63	6.25
R4b	4.09	1.22	11.09	7.47	10.83
R5a	7.94	2.37	7.80	142.38	7.01
R5b	3.99	1.19	33.02	5.19	7.72
R6a	29.65	8.85	10.05	170.05	9.02
R6b	3.96	1.18	89.51	2.00	5.33
**Aver. all active**	**9.95**	**2.97**	**24.19**	**58.51**	**10.16**
*Upper Silesia—the Anna coal waste dump*					
A1	9.70	2.89	41.21	5.23	46.87
A2	4.03	1.20	11.87	9.38	31.85
A3	5.23	1.56	358.00	1.16	2.54
A4	5.76	1.72	11.13	4.94	33.65
A5	5.66	1.69	2299.29	–	0.95
**Aver. all active**	**6.08**	**1.81**	**544.30**	**4.14**	**23.17**
*Upper Silesia—the Czerwionka-Leszczyny coal waste dump*					
CzL1	3.74	1.12	0.16	1.25	61.58
CzL2	4.28	1.28	44.91	72.29	4.59
CzL3	3.78	1.13	12.49	245.86	8.10
CzL4	3.90	1.16	23.57	55.22	10.80
CzL5	3.87	1.15	29.48	–	15.73
**Aver. all active**	**3.91**	**1.17**	**22.12**	**74.92**	**20.16**
*Lower Silesia—the Słupiec coal waste dump*					
S1a	5.11	1.53	19.25	0.48	12.94
S1b	5.63	1.68	376.54	1.32	6.61
S1c	5.63	1.68	376.54	1.32	6.61
S1d	14.26	4.26	29.82	5.52	9.24
S1e	31.94	9.53	32.57	9.74	22.27
S1f	28.81	8.60	45.89	11.26	22.05
S1g	26.24	7.83	79.98	6.22	7.27
S2a	24.90	7.43	21.70	4.36	24.99
S2b	29.85	8.91	15.11	5.23	33.35
S3	33.69	10.06	24.07	6.20	19.48
S4a	19.48	5.81	81.10	41.71	8.95
S4b	23.13	6.91	51.45	13.73	16.44
S5a	14.84	4.43	22.92	34.15	64.19
S6	4.48	1.34	9.21	–	9966.66
S7	21.52	6.42	23.16	0.91	682.29
S8a	19.86	5.93	91.87	13.80	7.83
S8b	28.87	8.62	60.51	10.23	13.42
S9	20.61	6.15	65.15	5.43	10.90
S10a	8.54	2.55	752.45	–	34.12
S10b	3.70	1.11	121.81	0.63	0.41
S11	3.72	1.11	0.00	3.66	0.36
**Aver. active S1**–**S10**	**18.55**	**5.54**	**115.06**	**8.61**	**548.50**
**Inactive S11**	**3.72**	**1.11**	**0.00**	**3.66**	**0.36**
*Lower Silesia—the Nowa Ruda and Przygórze coal waste dumps*					
N1	3.81	1.14	0.00	0.67	0.29
N2	4.36	1.30	102.26	0.33	5.12
N3	4.42	1.32	78.09	0.34	7.98
P1	3.85	1.15	42.87	0.29	2.16
**Aver. active N2 and N3**	**4.39**	**1.31**	**90.18**	**0.34**	**6.55**
**Aver. inactive N1, P1**	**3.83**	**1.14**	**21.44**	**0.48**	**1.22**
**US aver. active**	**7.68**	**2.29**	**153.72**	**35.46**	**81.41**
**US aver. inactive**	**4.43**	**1.32**	**1691.43**	**3.28**	**0.477**
**LS aver. active**	**11.47**	**3.42**	**102.62**	**4.47**	**277.53**
**LS aver. inactive**	**3.78**	**1.13**	**10.72**	**2.07**	**0.79**

Averages are shown in bold

^a^*OD* oxygen decrease. a ratio shows oxygen content decrease compared to the O_2_ content (vol.) in the atmospheric air; OD = 3.35 × N_2_/O_2_ content in a gas sample, where 3.35 is the value of atmospheric ratio of N_2_/O_2_ (vol.)

^b^Sat/UnsatHC = a ratio of a sum of all saturated C_2_–C_7_ hydrocarbons to a sum of all unsaturated hydrocarbons

^c^CH_4_/all HC = a ratio of methane content to a sum of all hydrocarbons

“–” Compounds were not found

1. Oxygen decrease (OD) calculated as N_2_/O_2_ ratio to the ratio of these same gases in the atmosphere (3.35; vol.: vol.). N_2_ is assumed to be inert; it neither reacts with coal waste nor is released. The OD value reflects O_2_ consumption during heating.
2. The ratio of saturated to unsaturated hydrocarbons (S/UnS). It is assumed that unsaturated hydrocarbons are the products of organic matter macromolecule cracking. This parameter reflects the thermal destruction of organic matter.
3. Carbon dioxide/methane, the ratio of two major components of gas emissions from coal waste dumps

### Oxygen decrease (OD) ratio

Oxygen decrease is caused by oxidation of organic matter due to self-heating. Thus, the OD value reflects the process intensity; the higher the value, the more intense the self-heating. OD values are arbitrarily designated as follows: 1.0–1.7 (low self-heating or none), 1.8–3.0 (moderate heating), > 3.0 (intense heating).

**Fig. 4:**
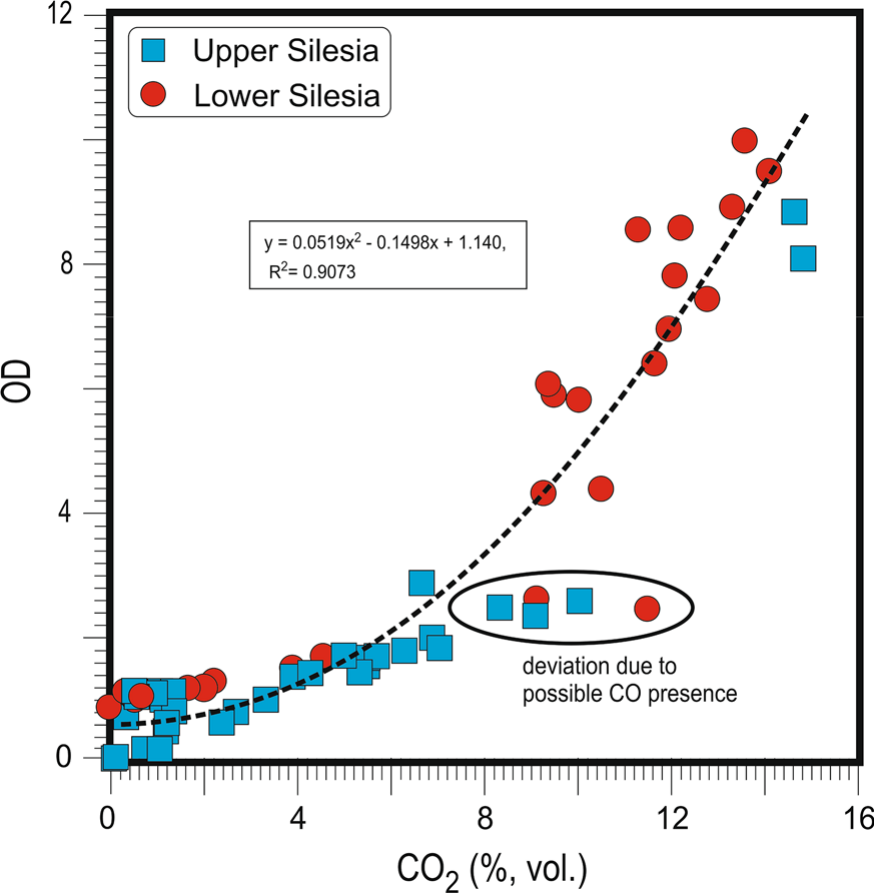
Correlation between oxygen decrease (OD) composition compared to atmospheric O_2_ content and content (rel.%) of CO_2_ in waste dump gas

Samples with very low OD values are W1, W2a, W4a, W7, W8, all Rymer Cones gases taken in the later series (R2–R4b, R5b, R6b), and A2–5, CzL1–5, S1a–c, S 6, S10b, S11, P1, and N1–3. Moderate values characterize only six samples, i.e., W3b, R1a and b, R5a, A1, and S10a. The highest values pertain to W2b, W3a, W4b, W5, W6a–b, R4a, R6a, S1d–g, S2–5, and S7–9 (Table 3). OD correlates with > 2.0% (vol.) contents of CO_2_ in the total gas composition; the substrate–product relationship is confirmed by inverse correlations (*r* = − 0.89 for US and − 0.98 for LS) of relative contents (vol.) of CO_2_ versus O_2_ and positive correlations (*r* = 0.87 for US and − 0.93 for LS) between OD values and CO_2_ relative contents (Fig. 4). The particularly high correlations (*r* = 1.00) between OD and CO_2_ for the Wełnowiec gas samples probably reflect firefighting activity; during sampling, the dump was opened to cool burning waste, increasing O_2_ access, and intensifying combustion and elevating temperatures (< 700 °C). The strong correlation indicates that most O_2_ was consumed by CO_2_ production with other oxides playing only a very minor role.

Higher OD values generally characterize sites with temperatures > 70 °C, the self-heating threshold temperature (Gumińska and Różański 2005). Below, only mild organic matter oxidation occurs. If the threshold is breached, a rapid further temperature increase leads to self-heating and, potentially, opens fire. Alternatively, slow cooling occurs and, in time, organic matter weathering. The initial stage of self-heating lasting several days is difficult to recognize; there are few external signs. However, it is revealed by elevated CO_2_ in dump gases and decreased O_2_ (Tabor 2002).

### Saturated to unsaturated hydrocarbons ratio (S/UnS)

Values of this parameter reflect the predominance of saturated hydrocarbons in all samples apart from W3b, W4a (~ 1.0), NR1–4, NRS1a, NRS7, and NRS10b (Table 3). At sites without thermal activity, unsaturated hydrocarbons were often absent. In others, the pattern is more complex as self-heating releases hydrocarbons of both types together. High S/UnS values as in W3c tend to be associated with the highest temperatures, as are higher OD values. The Rymer Cones gases sampled in 2011 and 2015 differ in their S/UnS values; the latter have lower values due to comparatively lesser expulsion of saturated hydrocarbons.

### Differences in self-heating activity and its dynamics between Upper and Lower Silesia coal waste dumps

There are three factors which should be considered as influencing gas composition: (1) temperature, (2) the stage of self-heating (initial, ongoing, or waning), and (3) characteristics of coal wastes organic matter and minerals. Differences in the chemistry of gas emitted from dumps in LS and US are related to all three factors, but their relative importance varies.

At the time of sampling, at the LS sites, only mild thermal activity prevailed with temperatures < 70 °C, i.e., below the threshold temperature above which intense self-heating begins (Sokol 2005). Thus, only mild oxidation of coal waste organic matter occurred there (Table S2). Pronounced self-heating in the US sites involved much higher temperatures and, as a result, gas production was much intense (Table S3). There are also distinctive differences between both basins in average temperatures measured at dump surfaces and subsurface in active and inactive sites. At the active US sites, the average temperature measured at the surface was 62 °C and subsurface 137 °C, whereas those measured in the active LS sites were 38 and 66 °C, respectively. At thermally inactive US sites, these temperatures were 2 and 7 °C and, for the LS, 21 and 29 °C, respectively.

It follows that gas composition in the thermally active LS sites is characteristic of waning self-heating, with OD values approaching 1.0 due to the low consumption of oxygen in the process. Methane is absent, or contents are very low. This is reflected by values of CH_4_/CO_2_ which, at the LS sites, are similar to those of the US inactive sites. S/UnS follows a similar pattern. Thus, gas composition seems to mainly reflect self-heating stage and temperature level, particularly whether the threshold temperature (60–80 °C) is exceeded or not (Sokol 2005; Pone et al. 2007).

However, correlations between CO_2_ and CH_4_ contents (Fig. 3) in the individual basins show a small difference, most probably caused by differences in the initial characteristics of the coal waste organic matter. The LS coals are of higher rank than the US coals (Zdanowski and Żakowa 1995). The organic matter of the adjacent waste rocks is likewise. This makes the LS coal waste organic matter more inert as labile aliphatic groups were expelled earlier during its natural maturation within the deposit and, thus, less prone to produce aliphatic compounds when heated. It also explains the slight shift in the proportions of CO_2_ and CH_4_ that reflects lower CH_4_ production and, thus, its rapid oxygenation to CO_2_ in the LS dumps. Moreover, this difference in organic maturity may explain why average concentrations of several dominant hydrocarbons (Table S3) are much higher in the active US sites, e.g., CH_4_ (× 6), C_2_–C_4_ saturates (× 2–7), propylene and acetylene (× 5) and H_2_ (× 8). The lower resistance and rank of the US organic matter are also reflected in more pronounced temperature effects on gas compositions in active and inactive sites, e.g., much higher contents of CH_4_ and C_2_H_6_ hydrocarbons (ca × 1400 and × 330, respectively) in US than in LS sites (ca × 20 and × 220, respectively).

### Gas composition and thermal activity stage

**Fig. 5:**
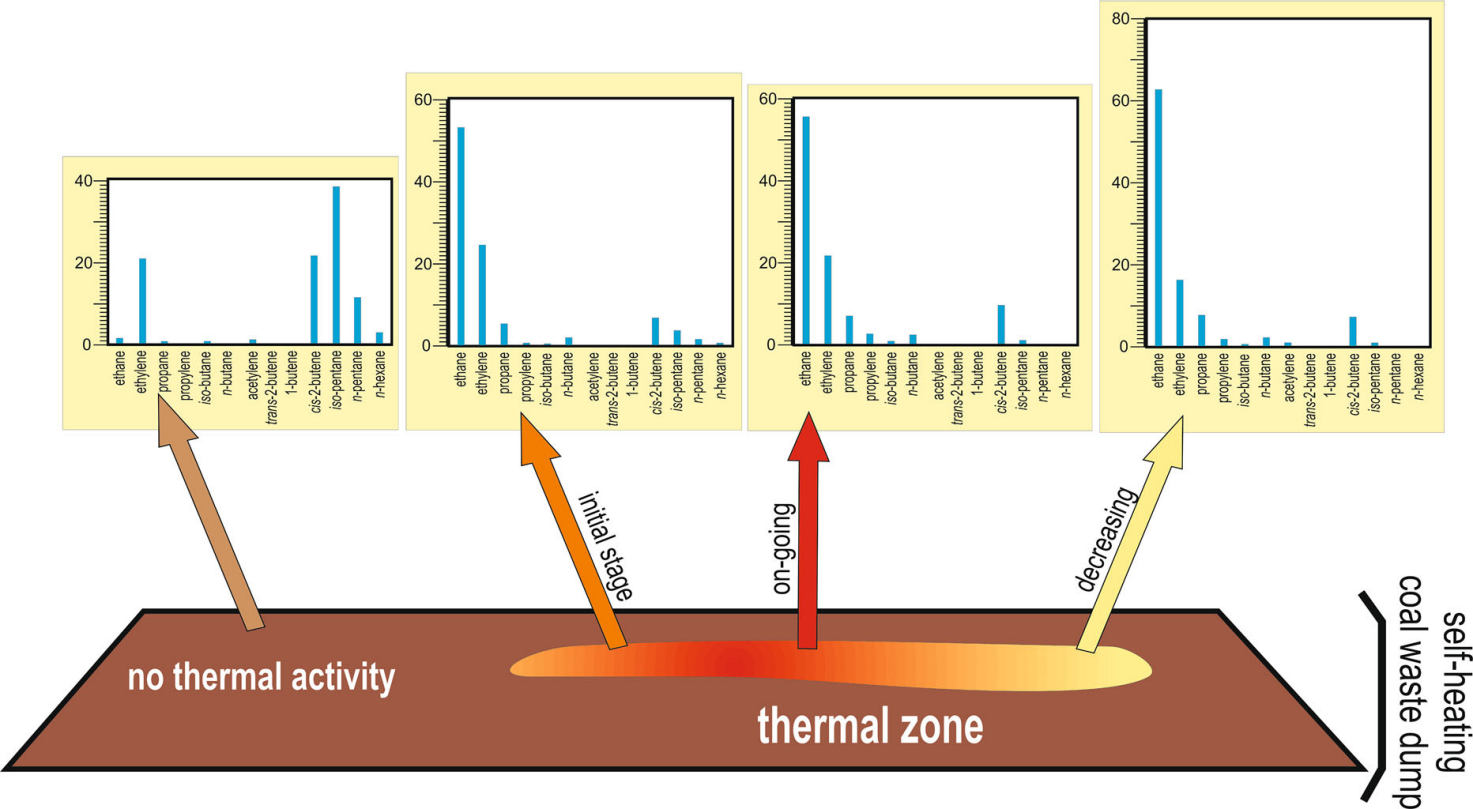
Representative averaged gas compositions in thermally inactive sites and the initial, ongoing, and waning self-heating stages in the Wełnowiec dump

**Fig. 6:**
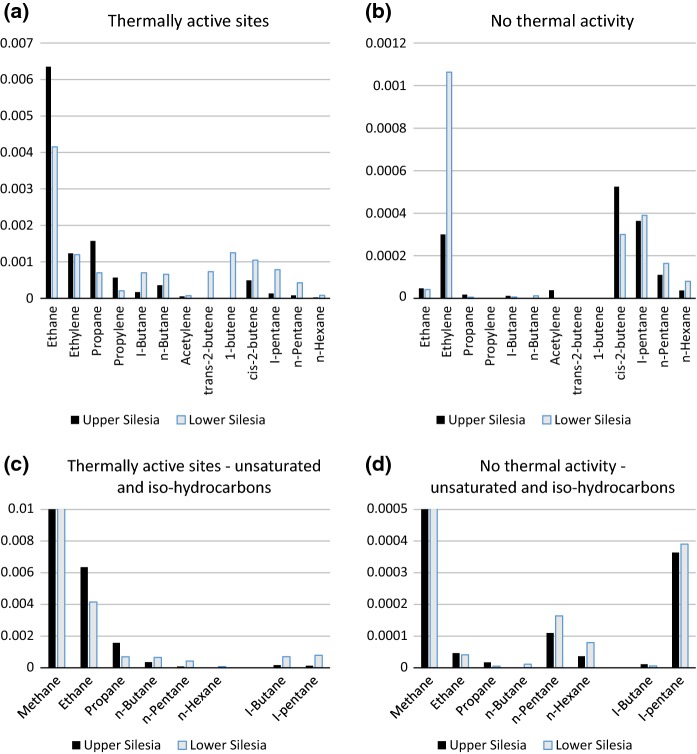
Comparison of gas distributions in thermally active sites and in sites with no thermal activity (rel.% of total gas composition) in the Upper and Lower Silesian basins

Whereas CH_4_ predominates at all thermally active sites, the compositions of heavier hydrocarbons, i.e., C_2_–C_7_, correlate better with self-heating stages (Figs. 5 and 6). At inactive sites, apart from atmospheric gases and elevated CO_2_, C_4_–C_6_ hydrocarbons and ethylene, possibly of biological origin, are dominant. Initial- and waning-stage gases have compositions similar to each other, with ethane being the predominant hydrocarbon. The ongoing, well-developed stage of self-heating with site temperatures > 70 °C is characterized by slightly higher emissions of C_3_–C_6_ hydrocarbons compared to the initial and waning stages, commonly heavier unsaturated hydrocarbons and H_2_. However, the likely impossibility of reliably differentiating heating stages on gas compositions alone underscores the value of thermal mapping.

### Health and environmental impact

Exceptionally high CO_2_ levels together with other gases emitted have adverse effects on health, particularly with whole-life exposure. It is difficult to assess how large the US and LS population is exposed to coal waste dump gases since the range of contaminant transport is unknown and most possibly affected by several factors, e.g., fire intensity, prevailing winds, and the dump architecture. Research on these problems is in its infancy. The total population of Upper Silesia is ca 4.599 million and that of Lower Silesia ca 2.910 million, with average densities of 373 and 146 person/km^2^, respectively (stat.gov.pl 2014). Densities are particularly high in the areas where ca 200 US and 130 LS coal waste dumps are located, i.e., 2000–3000 person/km^2^; communities clustered around the mines and associated smelters. For example, the Wełnowiec dump lies within 2.5 km of three Katowice districts, Koszutka, Bogucice, and Dąb, with 10000, 14000, and 7000 inhabitants, respectively. The Osiedle Tysiąclecia area slightly further away houses ca 21 000 inhabitants. The worst impacts probably affect settlements such as Skała, Bunczowiec, and Bułowiec (the Rydułtowy districts with ca 8000 inhabitants) located 300 m from the Szarlota dump, 500 m from the Anna-Pszów dump, and ca 2 km m from the Marcel and Rymer Cones dumps, or the Niedobczyce residential area (ca 12000 inhabitants) located 100–200 m from the Rymer Cones. Both regions are characterized by high degrees of citizens mobility to and from homes and working places every day which makes the real impact difficult to assess. However, the fact that incidences of lung cancer and other lung and cardiovascular illnesses generally are much higher in Silesia than elsewhere in Poland may be an additional indicator of exposure to self-heating pollutants (*Nowotwory*. 2013).

Greenhouse gas is probably the greatest concern as dump self-heating is not typically recognized as a significant source. Regrettably, awareness of the problem is low even in the scientific community, despite the worldwide occurrence of the phenomenon, e.g., Portugal, Australia, USA, China, and South Africa (e.g., Litchke 2005; Pone et al. 2007; Carras et al. 2009; Ribeiro et al. 2010; O’Keefe et al. 2010).

## Conclusions

Gas emissions from coal waste dumps in two coal mining basins in Poland are characterized by highly variable compositions with CO_2_ and CH_4_, major greenhouse gases predominating in all thermally active sites. Both CO_2_ and CH_4_ can greatly exceed values considered safe for health. The thermally active dumps should be regarded as their significant source. A strong substrate–product correlation between CO_2_ and relative percentage contents of CH_4_ points to CH_4_ oxidation to CO_2_ immediately after CH_4_ release during self-heating.

Gas emissions at inactive sites comprise CH_4_ and smaller amounts of C_3_–C_6_ hydrocarbons, mostly *n*-alkanes. Concentrations of CH_4_ at thermally inactive sites where fire had been extinguished or which were never burnt, exceed by several times atmospheric values. At these sites, CH_4_ is of a possible bacterial origin (as is ethylene) or reflects long-term leakage from rock pores. Even thermally inactive coal waste dumps should be deemed a long-term environmental hazard.

The main light hydrocarbons produced during self-heating are saturates. Their dominance over unsaturated hydrocarbons increases with temperature. Acetylene is rare and other alkynes were not found, possibly due to their higher chemical reactivity.

Oxygen decrease in the gases is mostly temperature-dependent with a threshold temperature of ca 70 °C. Whenever this level is reached, a significant decrease in oxygen content is registered. A strong substrate–product correlation between CO_2_ and O_2_ indicates that organic matter oxidation, not the formation of other oxides (including inorganic oxides), consumes most of the oxygen budget.

The distribution of heavier hydrocarbons in the dumps is influenced by the stage of self-heating attained. Initial and waning stages show similar gas compositions, whereas sites with ongoing self-heating show greater emission of heavier hydrocarbons, possibly related to higher temperatures. On a regional scale, the minor differences between emissions in the two Silesian coal basins are also mostly related to the self-heating stage pertaining or to differences in the thermal maturity of coal waste organic matter in both basins. The higher rank of LS organic matter makes it less prone to expelling hydrocarbons when heated. Critically, in Lower Silesia, self-heating is on the wane and most dumps already overburnt.

Since self-heating of coal waste dumps exposes large population in Upper and Lower Silesia, precautionary measures against any health dangers should be undertaken, e.g., monitoring of internal temperatures and initial-stage gases. The low threshold temperature (ca 70 °C) means that quick and relatively inexpensive cooling of the damp is possible before the beginning of intense self-heating. Otherwise, temperatures will increase rapidly up to ignition temperature over a few weeks. Unfortunately, it is not easily possible to dismantle coal waste dumps. Due to poor mechanical quality of Silesian coal wastes, their reuse is limited to overburnt material. To limit population exposure to harmful emissions, limiting access to dumps may be advisable, particularly those with ongoing heating.

## Electronic supplementary material

Below is the link to the electronic supplementary material.

Supplementary material 1 (DOCX 1068 kb)

Supplementary material 2 (DOCX 1314 kb)

Supplementary material 3 (DOCX 1288 kb)

Supplementary material 4 (DOCX 36 kb)

Supplementary material 5 (DOCX 14 kb)

Supplementary material 6 (DOCX 46 kb)

## References

[CR1] Association Advancing Occupational and Environmental Health (ACGIH), (1999). In *Threshold limit values for chemical substances and physical agents and biological exposure indices*, Cincinnati, OH.

[CR2] Barosz S (2003). Technical, economical and environmental conditions of management of coal-waste dumps using the mines from the Rybnik Coal District as examples.

[CR3] Borzęcki, R., & Marek, A. (2013). Geotourist attractions of the slag heap of the former coal-mine “Nowa Ruda”. In *Mining History-the part of European cultural heritage 5*. Wyd. Ofic. Wyd. PWroc. Wrocław, (pp. 15–25) (**in Polish with English abstract**).

[CR4] Brake DJ, Bate GP (1999). Criteria for the design of emergency refuge stations for an underground metal mine. Journal of the AusIMM.

[CR5] Broussard L, Levine B (1999). Inhalants. Principles of forensic toxicology.

[CR6] Carras JN, Day SJ, Saghafi A, Williams DJ (2009). Greenhouse gases emissions from low-temperature oxidation and spontaneous combustion at open-cut coal mines in Australia. International Journal of Coal Geology.

[CR7] Ciesielczuk J, Czylok A, Fabiańska MJ, Misz-Kennan M (2015). Plant occurrence on burning coal-waste—a case study from the Katowice-Wełnowiec dump, Poland. Environmental and Socio-economic Studies.

[CR8] Ciesielczuk J, Janeczek J, Cebulak S (2013). The cause and progress of the endogenous coal fire in the remediated landfill in the city of Katowice. Przegląd Geologiczny.

[CR9] Czechowski F, Marzec A, Czajkowska S (2007). Tworzenie się wodoru na drodze niskotemperaturowego utleniania węgla kopalnego tlenem z powietrza (Hydrogen formation upon low-temperature oxidation of bituminous coal with air oxygen, in Polish). Gospodarka Surowcami Mineralnymi.

[CR10] Davidi S, Grossman SL, Cohen H (1995). Organic volatile emissions accompanying the low-temperature atmospheric storage of bituminous coals. Fuel.

[CR11] Dlugokencky, E. (2016). Trends in atmospheric methane, Global greenhouse gas reference network, NOAA Earth System Research Laboratory, (www.esrl.noaa.gov/gmd/ccgg/trends_ch4/). Accessed 3 Sept 2017.

[CR12] EPA (Environmental Protection Agency), (2005). 25-05-2005/US-EPA/Methane/EP A-Home/Global Warming Home: accessed 17/12/2005: http:www.epa.gov/methane. Accessed 3 Sept 2017.

[CR13] Fabiańska MJ, Ciesielczuk J, Kruszewski Ł, Misz-Kennan M, Blake DR, Stracher G, Moszumańska I (2013). Gaseous compounds and efflorescences generated in self-heating coal-waste dumps–a case study from the Upper- and Lower Silesian Coal Basins (Poland). International Journal of Coal Geology.

[CR14] Finkelman RB (2004). Potential health impact of burning coal beds and waste banks. International Journal of Coal Geology.

[CR15] Frużyński A (2012). Hard coal mines in Poland.

[CR16] Grossman SL, Davidi S, Cohen H (1993). Molecular hydrogen evolution as a consequence of atmospheric oxidation of coal: Batch reactor simulation. Fuel.

[CR17] Grossman SL, Davidi S, Cohen H (1994). Emission of toxic and fire hazardous gases from open air coal stockpiles. Fuel.

[CR18] Gumińska J, Różański Z (2005). Analiza aktywności termicznej śląskich składowisk odpadów powęglowych (Analysis of thermal activity in Silesian coal-waste landfills, in Polish). Karbo.

[CR19] Hower JC, Henke K, O’Keefe JMK, Engle MA, Blake DR, Stracher GB (2009). The Tiptop coal-mine fire, Kentucky: Preliminary investigation of the measurement of mercury and other hazardous gases from coal-fire gas vents. International Journal of Coal Geology.

[CR20] Johnson PR, Ecker JR (1998). The ethylene gas signal transduction pathway: A molecular perspective. Annual Review of Genetics.

[CR21] Kaymakçi E, Didari V (2002). Relations between coal properties and spontaneous combustion parameters. Turkish Journal of Engineering and Environmental Sciences.

[CR22] Kędzior S (2009). Accumulation of coal-bed methane in the south-west part of the Upper Silesian Coal Basin (southern Poland). International Journal of Coal Geology.

[CR23] Killops SD, Killops VJ (2005). An introduction to organic geochemistry.

[CR24] Kim AG, Stracher GB (2007). Greenhouse gases generated in underground coal-mine fires. Geology of coal fires: Case studies from around the world.

[CR25] Korban Z (2011). Problem of mining waste and their impact on environment in the case of burrow no. 5A/W-1 of “x” mine. Górnictwo i Ekologia.

[CR26] Kotarba M (2001). Composition and origin of coalbed gases in the Upper Silesian and Lublin basins, Poland. Organic Geochemistry.

[CR27] Krishnaswamy S, Agarwal PK, Gunn RD (1996). Low-temperature oxidation of coal. 3. Modelling spontaneous combustion in coal stockpiles. Fuel.

[CR28] Krishnaswamy S, Bhat S, Gunn RD, Agarwal PK (1996). Low-temperature oxidation of coal. 1. Single–particle reaction–diffusion model. Fuel.

[CR29] Litschke, T. (2005) Innovative technologies for exploration, extinction and monitoring of coal fires in North China: detailed mapping of coal fire sites in combination with in situ flux measurements of combustion-gases to estimate gas flow balance and fire development (Wuda Coal Field, Inner Mongolia Autonomous Region). Unpublished PhD thesis, Dortmund, Universität Duisburg-Essen. http://www.coalfire.caf.dlr.de/media/download/results/Diplomarbeit-Litschke.pdf. Accessed on 29 March 2011.

[CR30] Liu Ch, Li S, Qiao Q, Wang J, Pan Z (1998). Management of spontaneous combustion in coal mine waste tips in China. Water, Air, and Soil pollution.

[CR31] Misz-Kennan, M., Ciesielczuk, J., Tabor, A. (2013) coal-waste dump fires of Poland. (Chapter 15) In Stracher, G. B., Prakash, A., Sokol, E. V. (Eds.), 2013. *Coal and peat fires: A global perspective*, Volume 2: Photographs and Multimedia Tours, Amsterdam: Elsevier, (pp. 233–311) (ISBN: 0978-0-444-59412-9).

[CR32] Nádudvari Á (2014). Thermal mapping of self-heating zones on coal-waste dumps in Upper Silesia (Poland)—a case study. International Journal of Coal Geology.

[CR33] Nádudvari Á, Fabiańska MJ (2016). Use of geochemical analysis and vitrinite reflectance to assess different self-heating processes in coal-waste dumps (Upper Silesia, Poland). Fuel.

[CR34] Nagahama K, Ogawa T, Fuji T, Fukuda H (1992). Classification of ethylene-producing bacteria in terms of biosynthetic pathways to ethylene. Journal of Fermentation and Bioengineering.

[CR35] Nowotwory złośliwe w województwie śląskim (Malicious cancers in Silesia District), (2013). (Ed.) Śląski Urząd Wojewódzki w Katowicach Wydział Nadzoru nad Systemem Opieki Zdrowotnej Oddział Analiz i Statystyki Medycznej, (pp. 81).

[CR36] O’Keefe JMK, Hanke KH, Hower JC, Engle MA, Stracher GB, Stucker JD, Drew JW, Staggs WD, Murray TM, Hammond ML, Adkin KD, Mullins BJ, Lemley EW (2010). CO_2_, CO, and Hg emissions from the Truman Shepherd and Ruth Mullins coal fires, eastern Kentucky, USA. Science of the Total Environment.

[CR37] Parafiniuk J, Kruszewski Ł (2010). Minerals of the ammonioalunite–ammoniojarosite series formed on a burning coal dump at Czerwionka, Upper Silesian Coal Basin. Poland. Mineral Magazine.

[CR38] Pauluhn J (2016). Risk assessment in combustion toxicology: Should carbon dioxide be recognized as a modifier of toxicity or separate toxicological entity?. Toxicology Letters.

[CR39] Peischl J, Ryerson TB, Aikin KC, de Gouw JA, Gilman JB, Holloway JS, Lerner BM, Nadkarni R, Neuman JA, Nowak JB, Trainer M, Warneke CD, Parrish D (2015). Quantifying atmospheric methane emissions from the Haynesville, Fayetteville, and northeastern Marcellus shale gas production regions. Journal of geophysical Research: Atmospheres.

[CR40] Peters V, Conrad R (1995). Methanogenic and other strictly anaerobic bacteria in desert soil and other oxic soils. Applied and Environmental Microbiology.

[CR41] Pone JDN, Hein KAA, Stracher GB, Annegarn HJ, Finkelman RB, Blake DR, McCormack JK, Schroeder P (2007). The spontaneous combustion of coal and its by-products in the Witbank and Sasolburg coalfields of South Africa. International Journal of Coal Geology.

[CR42] Prakash A, Schaefer K, Witte WK, Collins K, Gens R, Goyette MP (2011). A remote sensing and GIS based investigation of a boreal forest coal fire. International Journal of Coal Geology.

[CR43] Querol X, Izquierdo M, Monfort E, Alvarez E, Font O, Moreno T, Alastuey A, Zhuang X, Lu W, Wang Y (2008). Environment characterization of burnt coal gangue banks at Yangquan, Shanxi Province, China. International Journal of Coal Geology.

[CR44] Querol X, Zhuang X, Font O, Izquierdo M, Alastuey A, Castro I, van Drooge BL, Moreno T, Grimalt JO, Elvira J, Cabañas M, Bartroli R, Hower JC, Ayora C, Plana F, López-Soler A (2011). Influence of soil cover on reducing the environmental impact of spontaneous coal combustion in coal-waste gobs: A review and new experimental data. International Journal of Coal Geology.

[CR45] Ribeiro J, Ferreira, da Silva E, Flores D (2010). Burning of coal-waste piles from Douro Coalfield (Portugal): Petrological, geochemical and mineralogical characterization. International Journal of Coal Geology.

[CR46] Saavedra J, Merino L, Kafarov V (2013). Determination of the gas composition effect in carbon dioxide emission at refinery furnaces. Chemical Engineering Transactions.

[CR47] Schneising O, Burrows JP, Dickerson RR, Buchwitz M, Reuter M, Bovensmann H (2014). Remote sensing of fugitive methane emissions from oil and gas production in North American tight geologic formations. Earth’s Future.

[CR48] Singh AK, Singh RVK, Singh M, Chandra H, Shukla NK (2007). Mine fire gas indices and their application to Indian underground coal mine fires. International Journal of Coal Geology.

[CR49] Skręt U, Fabiańska MJ, Misz-Kennan M (2010). Simulated water-washing of organic compounds from self-heated coal-wastes of the Rymer Cones Dump (Upper Silesia Coal Region, Poland). Organic Geochemistry.

[CR50] Sokol EV, Lepezin GG (2005). High-temperature processes of organic fuel decomposition as a thermal source for pyrometamorphic transformations. Combustion metamorphism.

[CR51] Speight, J.G. (2014). *The chemistry and technology of petroleum*, 5th Edition. Chemical Industries. CRC Group, Taylor & Francis Group, (pp. 1–928).

[CR52] Stracher GB, Taylor TP (2004). Coal fires burning out of control around the world: Thermodynamic recipe for environmental catastrophe. International Journal of Coal Geology.

[CR53] Tabor, A. (2002). Monitoring of coal-waste dumps, re-cultivated dumps and other collection sites of Carboniferous waste rocks in the light of many years experience. In *VII Conference “Long term proecological undertakings in the Rybnik Coal Area”, Rybnik*, (pp. 131–141) (**in Polish**).

[CR54] Tetzlaff, A. (2004). Coal Fire quantification using ASTER, ETM and BIRD satellite instrument data. PhD thesis Ludwig Maximilians University, Munich, Germany, (pp. 2–148), http://edoc.ub.uni-muenchen.de/4398/1/tetzlaff_anke.pdf. Accessed 4 July 2018.

[CR55] Tung HC, Bramall NE, Price PB (2005). Microbial origin of excess methane in glacial ice and implications for life on Mars. Proceedings of the National Academy of Sciences.

[CR56] www.ogimet.com.

[CR57] www.landsat.usgs.gov/what-are-band-designations-landsat-satellites.

[CR58] www.stat.gov.pl, (2014).

[CR59] Xie J, Xue Sh, Cheng W, Wang G (2011). Early detection of spontaneous combustion of coal in underground coal mines with development of an ethylene enriching system. International Journal of Coal Geology.

[CR60] Yan R, Zhu H, Zheng Ch, Xu M (2003). Emissions of organic hazardous air pollutants during Chinese coal combustion. Energy.

[CR61] Younger, P.L. (2004). Environmental impacts of coal mining and associated wastes: a geochemical perspective. In: R. Gieré, P. Stille, (Eds.) *Energy, waste and the environment: a geochemical perspective,* Geol. Soc. Spec. Publ. 236, (pp. 169–209).

[CR62] Zdanowski, A., & Żakowa H. (1995). The Carboniferous system in Poland. Works of Polish Geological Institute, (p. 215).

[CR63] Zhang J, Kuenzer C (2007). Thermal surface characteristics of coal fires 1: Results of in situ measurements. Journal of Applied Geophysics.

