# Supplementary figures and images for: Environmental influence of gaseous emissions from self-heating coal waste dumps in Silesia, Poland
Source: Environ Geochem Health. 2018 Jul 24;41(2):575–601. doi: 10.1007/s10653-018-0153-5 (PMC6510838; doi:10.1007/s10653-018-0153-5)

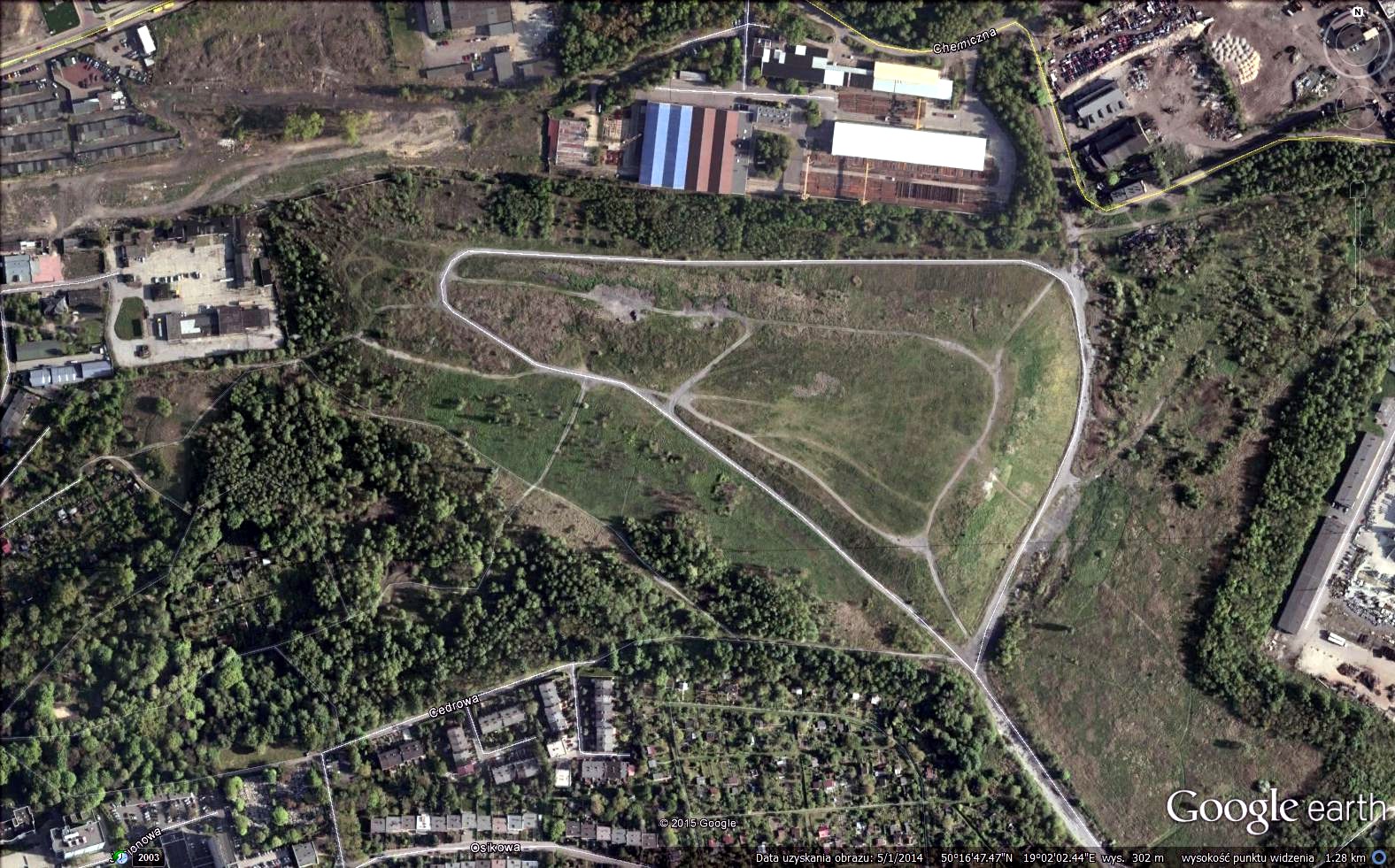


**W7**

**W8**

**W3**

**W2**

**W6**

**W4**

**W5**

**W1**

a


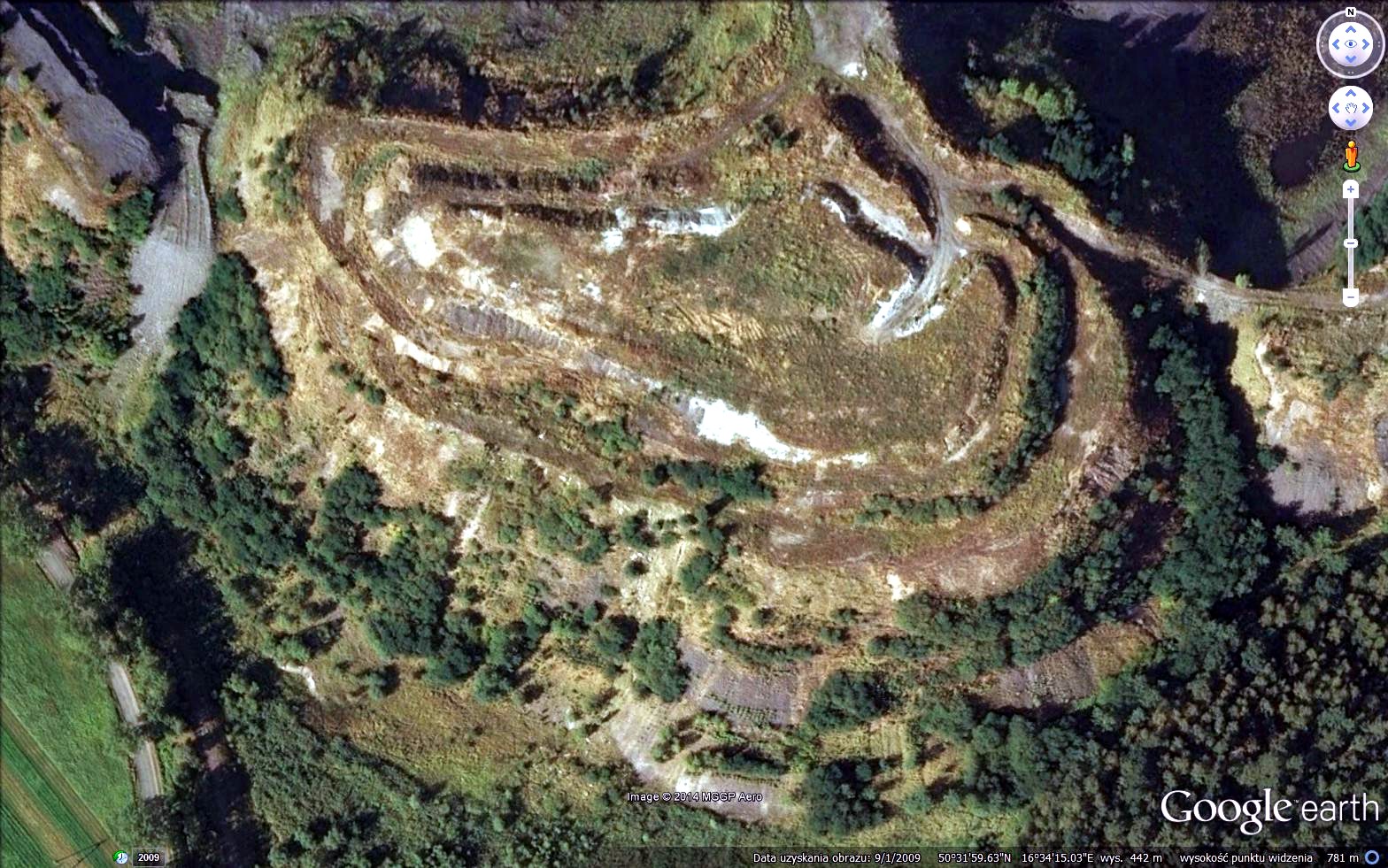


**1e-g**

**1a-d**

**5**

**4**

**10**

**2**

**3**

**9**

**6**

**7**

**8**

b

Supplement: Supplementary file 1 — Supplementary material 1 (DOCX 1068 kb) [file 10653_2018_153_MOESM1_ESM.docx]

| 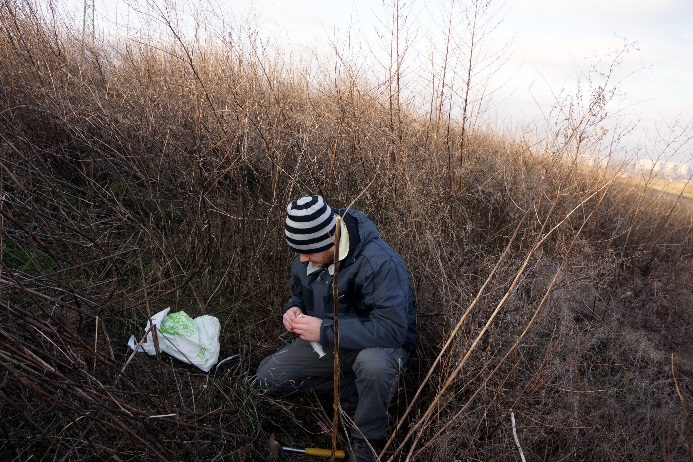  **W1** | 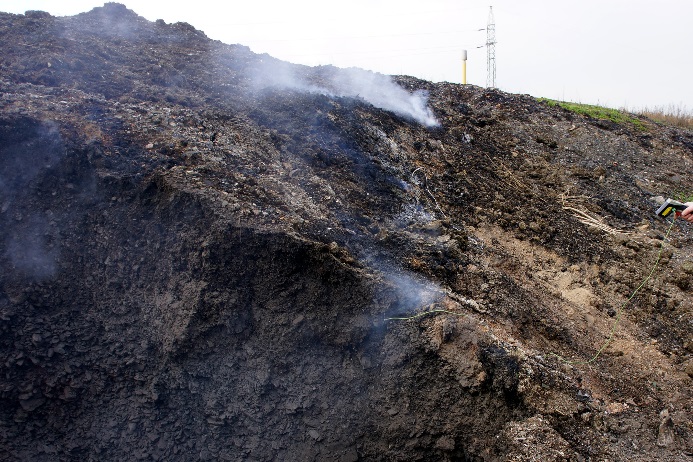  **W3a** |
| --- | --- |
| a | b |
| 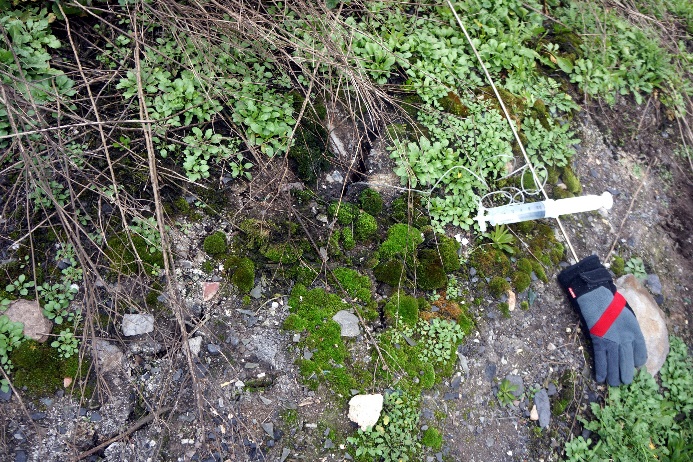  **W6** | 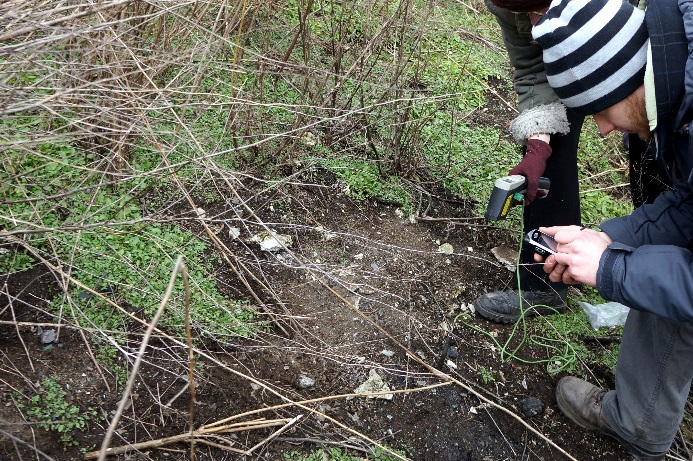  **W7** |
| c | d |
| 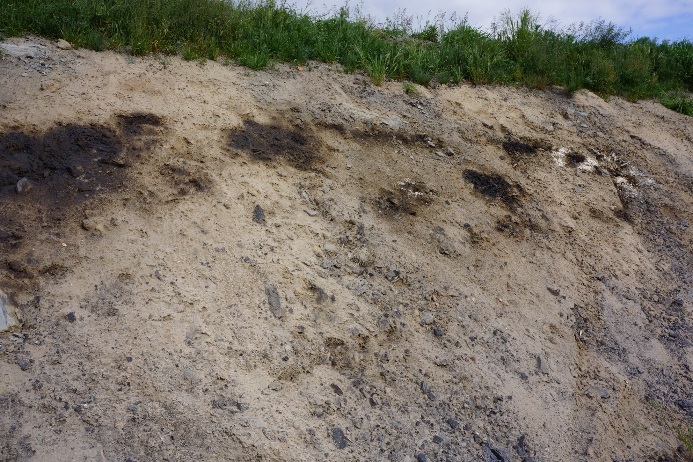 | 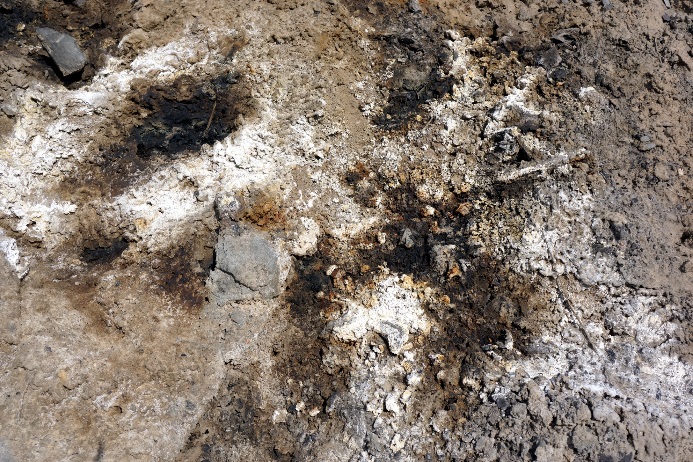 |
| e |  |

Supplement: Supplementary file 2 — Supplementary material 2 (DOCX 1314 kb) [file 10653_2018_153_MOESM2_ESM.docx]

| 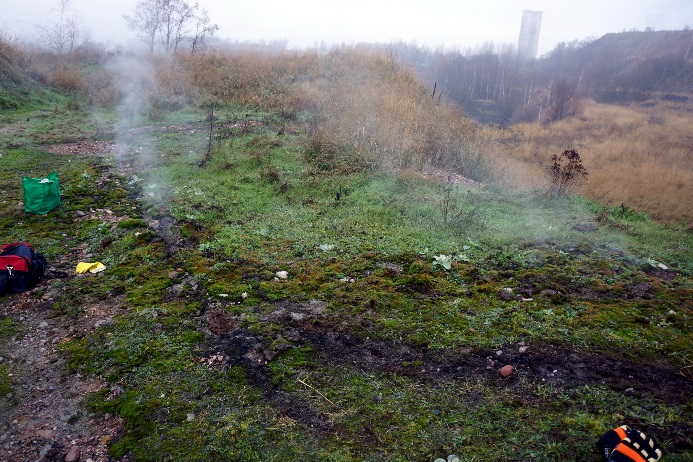  **S1b, c**  **S1a** | 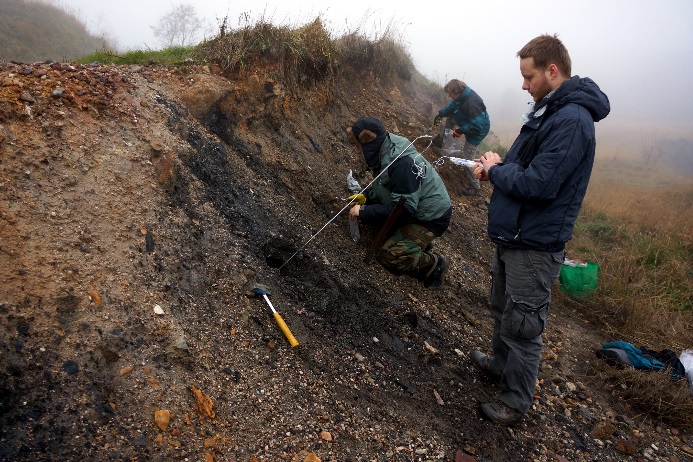  **S1g**  **S1e**  **S1f** |
| --- | --- |
| a | b |
| 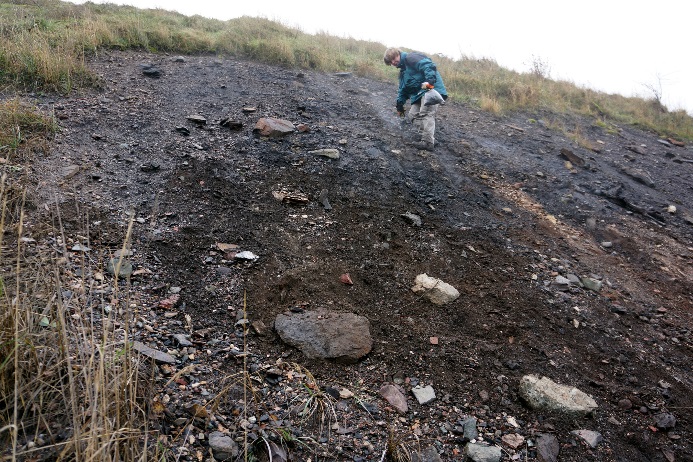  **S4a, b** | 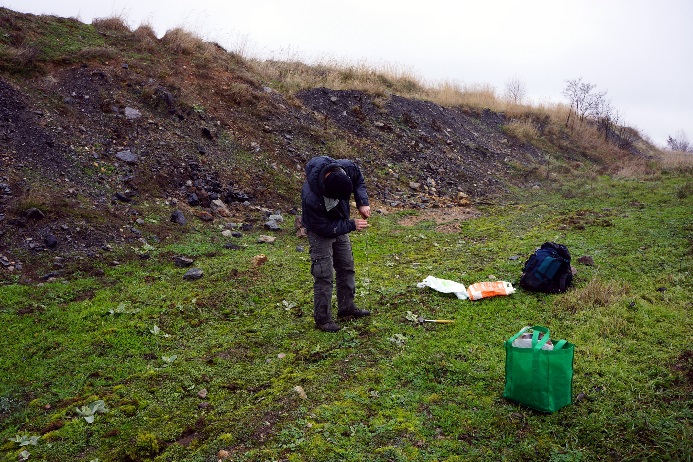  **S8a, b** |
| c | d |
| 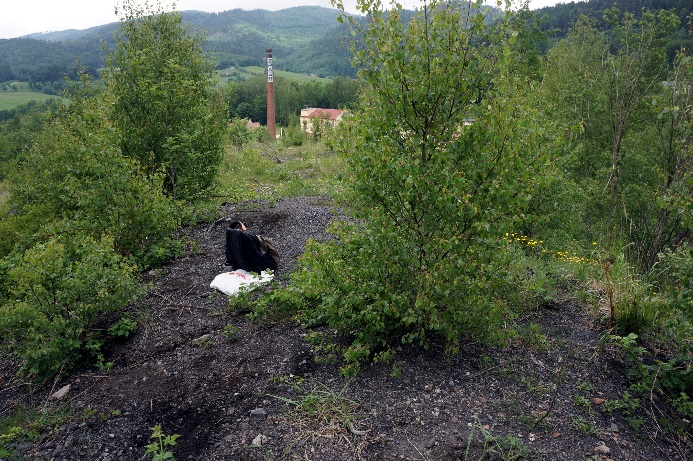  **P1** | 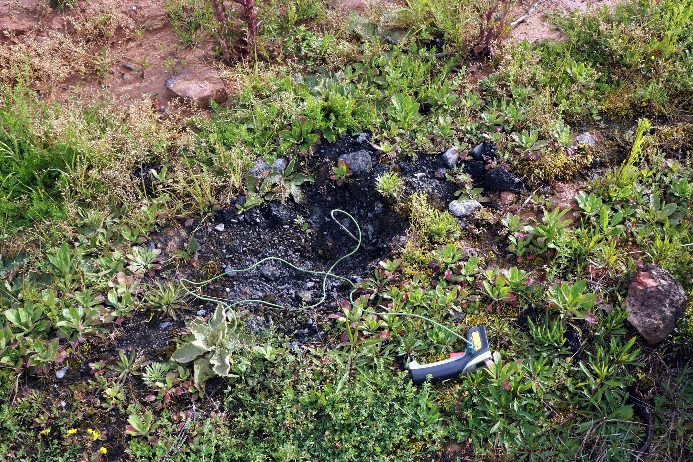  **N2** |
| e | f |

Supplement: Supplementary file 3 — Supplementary material 3 (DOCX 1288 kb) [file 10653_2018_153_MOESM3_ESM.docx]
