# Supplementary material for: Environmental influence of gaseous emissions from self-heating coal waste dumps in Silesia, Poland
Source: Environ Geochem Health. 2018 Jul 24;41(2):575–601. doi: 10.1007/s10653-018-0153-5 (PMC6510838; doi:10.1007/s10653-018-0153-5)
Supplement: Supplementary file 4 — Supplementary material 4 (DOCX 36 kb) [file 10653_2018_153_MOESM4_ESM.docx]

Table 1S.

| Sample code | x | y | z [m n.p.m.] | Temperature on surface [^o^C] | Temperature 0.3 m subsurface [^o^C] | Depth  of gas collection | Efflorescences | Thermal activity |
| --- | --- | --- | --- | --- | --- | --- | --- | --- |
| **Upper Silesia - the Wełnowiec coal waste dump** | | | | | | | | |
| W1 | 50^o^16’51.8 | 19^o^01’55.1 | 311 | 0 | 8 | 1 m |  | no |
| W2a | 50^o^16’51.7 | 19^o^01’55.7 | 310 | 80 | 430 | 3 cm | organic efflorescences,  sal ammoniac, sulphur, bitumens | initial |
| W2b |  |  |  |  |  | 0.9 m |  | initial |
| W3a | 50^o^16’51.3 | 19^o^01’59.4 | 305 | 280 | 340 | 0.2 m | organic efflorescences,  sal ammoniac, sulphur, bitumens | initial |
| W3b |  |  |  |  |  | 1.5 m |  | initial |
| W4a | 50^o^16’50.8 | 19^o^02’03.5 | 310 | 36 | 430 | 3 cm | lack of efflorescences, bitumens seepage | on-going |
| W4b |  |  |  |  |  | 1 m |  | on-going |
| W5 | 50^o^16’50.3 | 19^o^02’02.8 | 310 | 33 |  | chimney | H_2_S | decreasing |
| W6a | 50^o^16’50.9 | 19^o^02’02.1 | 311 | 36 | 72 | 3 cm | H_2_S | decreasing |
| W6b |  |  |  |  |  | 1 m |  | decreasing |
| W7 | 50^o^16’44.2 | 19^o^02’13.9 | 297 | 2 | 8 | 0.4 m | lack of efflorescences | no |
| W8 | 50^o^16’43.8 | 19^o^02’11.0 | 300 | 3 | 6 | 0.5 m | lack of efflorescences | no |
| **Upper Silesia - the Rymer Cones coal waste dump** | | | | | | | | |
| R1a  R1b | 50^o^03’25 | 18^o^29’42 | 301 | 5 | 60 | 0.3 | gypsum, bitumens | on-going |
| R2 | 50^o^03’20 | 18^o^29’37 | 297 | 12 | 50 | 0.3 | gypsum, bitumens | on-going |
| R3 | 50^o^03’25 | 18^o^29’39 | 295 | 16 | 28 | 0.3 | gypsum, bitumens | on-going |
| R4a  R4b | 50^o^03’24 | 18^o^29’42 |  | 30 | 95 | 0.3 | lack of efflorescences, bitumens seepage | on-going |
| R5a  R5b |  |  |  | 25 | 105 | 0.3 | gypsum, bitumens | on-going |
| R6a  R6b |  |  |  | 50 |  | 0.3 | gypsum, sulphur, bitumens | on-going |
| **Upper Silesia - the Anna coal waste dump** | | | | | | | | |
| A1 | 18°25’20,39” | 50°02’41,06” | 290 | 150 | 300 | 60 | sulphur, bitumens | on-going |
| A2 | 18°25’18,59” | 50°02’44” | 317 | 54 | 72 | 80 |  | decreasing |
| A3 | 18°25’18,98” | 50°02’45,15” | 317 | 70 | 85 | 125 |  | decreasing |
| A4 | 18°25’20,57” | 50°02’42,58” | 317 | 70 | 82 | 60 | sulphur, bitumens | on-going |
| A5 | 18°25’18,67” | 50°02’41,71” | 317 | 75 | 90 | 100 |  | decreasing |
| **Upper Silesia - the Czerwionka-Leszczyny coal waste dump** | | | | | | | | |
| CzL1 | 18°40’44,77” | 50°09’32,8” | 338 | 30 | 50 | 100 | lack of efflorescences, bitumens seepage | on-going |
| CzL2 | 18°40’45,93” | 50°09’34,34” | 348 | 60 | 80 | 100 | lack of efflorescences, bitumens seepage | on-going |
| CzL3 | 18°40’45,57” | 50°09’34,46” | 349 | 65 | 85 | 100 | sulphur, mascagnite, boussingaultite, bitumens, traces of alunite and sylvine | on-going |
| CzL4 | 18°40’45,17” | 50°09’34,68” | 352 | 70 | 90 | 70 | bitumens | on-going |
| CzL5 | 18°40’46,06” | 50°09’34,91” | 352 | 24 | 55 | 80 | bitumens | on-going |
|  | | | | | | | | |
| **Lower Silesia - the Słupiec coal waste dump** | | | | | | | | |
| S1a | 50^o^32’01” | 16^o^34’17” | 506 | 50 | 75 | 0.9 m | gypsum, bitumens, traces of alunite-(Na) | on-going |
| S1b |  |  |  | 50 | 65 | 0.05 m | traces of rosenite, gypsum, sulphur, bitumens | on-going |
| S1c |  |  |  | 50 | 65 | 1 m |  | on-going |
| S1d |  |  |  | 8 | 31 | ? m | sulphur, bitumens | on-going |
| S1e |  |  | 505 | 30 | 60 | 1 m | gypsum, mascagnite, sal ammoniac, mohrite-boussingaultite, bitumens | on-going |
| S1f |  |  |  | 25 | 54* | 0.4 m | gypsum, sulphur, bitumens, hydronium jarosite, tamarugite?, starkeyite? | on-going |
| S1g |  |  |  | 54 | 83 | 0.3 m | apjonite-pickeringite-halotrichite, gypsum, bitumens, traces of melanterite, mikasaite, tschermigite, hexahydrite | on-going |
| S2a | 50^o^32’02”406 | 16^o^34’10” | 499 | 35 | 80 | 0.9 m |  | on-going |
| S2b |  |  |  | 35 | 80 | 0.05 m | sulphur, bitumens, traces of gypsum | on-going |
| S3 |  |  |  | 11 | 28 | 0.5 m | lack of efflorescences | on-going |
| S4a | 50^o^32’02” | 16^o^34’11” | 496 | 22 | 70# | 1 m | sulphur, moganite SiO_2_, bitumens | on-going |
| S4b |  |  |  | 26 | 76 | 0.8 m |  | on-going |
| S5 | 50^o^32’02” | 16^o^34’11” | 482 | 75 | 85 | 0.9 m | gypsum, bitumens | on-going |
| S6 | 50^o^32’02”276 | 16^o^34’12”636 | 506 | 11 | 36* | 0.3 m | lack of efflorescences | on-going |
| S7 | 50^o^31’58”788 | 16^o^34’16”836 | 501 | 50 | 73 | 1 m | copiapite | on-going |
| S8a | 50^o^31’59”088 | 16^o^34’19”878 | 495 | 60 | 88 | 0.05 m | mohrite-boussingaultite, gypsum, sulphur, traces of alunite-(K)? | on-going |
| S8b |  |  |  | 60 | 88 | 0.9 m |  | on-going |
| S9 | 50^o^32’00”822 | 16^o^34’09”294 | 477 | 40 | 69 | 0.9 m | lack of efflorescences, bitumens seepage | on-going |
| S10a | 50^o^32’02”970 | 16^o^34’06”018 | 487 | 30 | 63$ | 0.3 m | aragonite, calcite, hydromagnesite | on-going |
| S10b |  |  |  | 23 | 29* | 0.2 m |  | on-going |
| S11 | 50^o^32’08”364 | 16^o^34’18”570 | 510 | 3 | 5 | 1 m | lack of efflorescences | no |
| **Lower Silesia - the Nowa Ruda coal waste dump** | | | | | | | | |
| N1 | 50^o^35’05”7 | 16^o^31’0.7 | 521 | 40 | 60* | 0.2 | lack of efflorescences | decreasing |
| N2 |  |  |  |  |  | 1.2 |  | decreasing |
| N3 | 50^o^35’04.4 | 16^o^31’07.4 | 516 | 55 | 80-100 | 0.1 | lack of efflorescences | decreasing |
| **Lower Silesia - the Przygórze coal waste dump** | | | | | | | | |
| P1 | 50^o^36’04.6 | 16^o^33’32.7 | 532 | 21 | 21 | 0.1 | lack of efflorescences | no |

* - temperature 0.2 m subsurface # - temperature 0.15 m subsurface $ - temperature 0.1 m subsurface
