# Supplementary material for: Environmental influence of gaseous emissions from self-heating coal waste dumps in Silesia, Poland
Source: Environ Geochem Health. 2018 Jul 24;41(2):575–601. doi: 10.1007/s10653-018-0153-5 (PMC6510838; doi:10.1007/s10653-018-0153-5)
Supplement: Supplementary file 5 — Supplementary material 5 (DOCX 14 kb) [file 10653_2018_153_MOESM5_ESM.docx]

| Dates | **Jelenia Góra** | | | **Kłodzko** | | | **Racibórz** | | | **Katowice** | | |
| --- | --- | --- | --- | --- | --- | --- | --- | --- | --- | --- | --- | --- |
|  | Lower Silesian meteorological stations | | | | | | Upper Silesian meteorological stations | | | | | |
|  | max | min | aver. | max | min | aver. | max | min | aver. | max | min | aver. |
| 26.02.1987 | 0.6 | -10.5 | -5.3 | 1.0 | -9.6 | -4.3 | - | - | - | - | - | - |
| 01.04.1993 | - | - | - | - | - | - | 7.1 | -4.0 | 1.7 |  |  |  |
| 22.12.2000 | -0.3 | -13.5 | -7.2 | -2.6 | -8.8 | -5.4 | - | - | - | - | - | - |
| 26.02.2001 | - | - | - | - | - | - | 1.9 | -18 | -7.8 | 1.0 | -14.2 | -6.5 |
| 09.12.2001 | -0.4 | -10.1 | -5.5 | -3.5 | -9.9 | -7.3 | - | - | - | - | - | - |
| 23.02.2003 | 7.0 | -13.8 | -5.7 | -0.7 | -10.4 | -6.8 | - | - | - | - | - | - |
| 25.01.2004 | -3.6 | -24.0 | -13.7 | -11.6 | -23.4 | -17.6 | - | - | - | - | - | - |
| 06.03.2004 | - | - | - | - | - | - | 0.2 | -18.2 | -7.4 | -1.0 | -17.4 | -9.2 |
| 04.12.2010 | - | - | - | - | - | - | -5.1 | -15.3 | -8.4 | -3.9 | -12.5 | -6.5 |
| 29.01.2011 | - | - | - | - | - | - | -0.1 | -9.6 | -5.1 | 0.2 | -11.1 | -5.1 |
| 14.04.2011 | - | - | - | - | - | - | 8.9 | 3.2 | 5.0 | 7.6 | 3.1 | 4.7 |
| 31.01.2012 | -7.8 | -14.0 | -11.3 | -9.0 | -16.4 | -13.5 | - | - | - | - | - | - |
| 11.02.2013 | - | - | - | - | - | - | -2.1 | -3.3 | -2.7 | -2.6 | -3.6 | -3.0 |
| 13.12.2013 | 5.6 | -6.2 | -2.5 | 3.3 | -1.2 | 1.4 | - | - | - | - | - | - |
| 06.03.2013 | 12.4 | -5.1 | 2.9 | 10.8 | 1.7 | 6.5 | - | - | - | - | - | - |
| 13.01.2014 | - | - | - | - | - | - | 5.1 | -2.5 | 1.8 | 4.1 | 0.9 | 2.8 |
| 28.05.2014 | 20.8 | 13.7 | 16.1 | 20.6 | 13.1 | 15.9 | - | - | - | - | - | - |
| 17.02.2015 | - | - | - | - | - | - | 3.6 | -4.7 | -1.9 | 2.5 | -6.2 | -2.7 |
| 07.02.2015 | 2.2 | -10.9 | -3.4 | 1.8 | -7.5 | -2.8 | - | - | - | - | - | - |
| 16.03.2015 | - | - | - | - | - | - | 11.0 | 2.6 | 6.8 | 11.4 | 2.2 | 6.2 |
| 12.11.2016 | - | - | - | - | - | - | 4.9 | 0.6 | 1.8 | 5.4 | 0.3 | 1.7 |
| 29.01.2017 | - | - | - | - | - | - | 1.7 | -9.8 | -5.5 | -0.9 | -14.6 | -8.0 |

Table S2 Temperatures (°C) from meteorological stations in Lower- and Upper Silesia pertinent to the creation of data from Landsat images and surface temperature measurements on Table 1. Source: [www.ogimet.com](http://www.ogimet.com)
