# Supplementary material for: Environmental influence of gaseous emissions from self-heating coal waste dumps in Silesia, Poland
Source: Environ Geochem Health. 2018 Jul 24;41(2):575–601. doi: 10.1007/s10653-018-0153-5 (PMC6510838; doi:10.1007/s10653-018-0153-5)
Supplement: Supplementary file 6 — Supplementary material 6 (DOCX 46 kb) [file 10653_2018_153_MOESM6_ESM.docx]

Table S3

| **Sample code** | **Methane** | **Ethane** | **Ethylene** | **Propane** | **Propylene** | **I-Butane** | ***n-*Butane** | **Acetylene** | ***trans-*2-Butene** | **1-Butene** | ***cis*-2-Butene** | ***i*-Pentane** | ***n*-Pentane** | ***n*-Hexane** | **Hydrogen** | **Carbon Dioxide** | | **Oxygen** | **Nitrogen** |
| --- | --- | --- | --- | --- | --- | --- | --- | --- | --- | --- | --- | --- | --- | --- | --- | --- | --- | --- | --- |
| mg/m^3^ | | | | | | | | | | | | | | | | (g/m^3^) | | | |
| Upper Silesia - the Wełnowiec coal waste dump | | | | | | | | | | | | | | | | | | | |
| W1 | 4.593 | 1.872 | 5.727 | 1.009 | - | 0.877 | - | 1.312 | - | - | 39.429 | 2.196 | - | - | - | | 63.95009 | 202.9040 | 1032.097 |
| W2a | 461.292 | 70.844 | 14.04 | 12.157 | 3.519 | 1.622 | 4.788 | - | - | - | 26.59 | 4.463 | - | - | 19.626 | | 51.73234 | 211.4263 | 1031.265 |
| W2b | 603.435 | 95.172 | 19.983 | 20.129 | 4.760 | 2.673 | 11.514 | - | - | - | 35.276 | 4.194 | 4.904 | - | 37.674 | | 162.6616 | 76.64493 | 1078.101 |
| W3a | 8993.000 | 301.114 | 27.65 | 18.269 | 6.137 | 1.613 | 4.249 | - | - | - | 41.004 | 2.967 | - | - | 2227.834 | | 134.7865 | 98.89031 | 1031.088 |
| W3b | 1677.426 | 486.716 | 28.002 | 93.876 | 0.866 | 7.913 | 32.622 | - | - | - | 27.694 | 7.404 | 6.171 | - | 28.455 | | 103.3883 | 128.7167 | 1068.070 |
| W3c | 534.187 | 27.245 | 10.008 | 4.477 | 3.403 | - | 2.947 | - | - | - | 35.462 | - | - | - | - | | 44.74157 | 255.4295 | 997.3908 |
| W4a | 2427.865 | 10.209 | 6.773 | - | - | - | - | - | - | - | - | 18.871 | 5.694 | - | 788.087 | | 123.0525 | 86.74864 | 1080.978 |
| W4b | 59392.550 | 606.477 | 44.332 | 97.342 | 15.58 | 17.308 | 35.978 | 12.485 | 5.522 | 4.288 | 46.985 | 12.066 | 8.97 | 2.693 | 62.72 | | 112.278 | 94.25727 | 991.136 |
| W5 | 46975.28 | 43.100 | 18.992 | 7.304 | 2.147 | 1.199 | 2.898 | - | - | - | 9.845 | 2.638 | - | - | - | | 109.8969 | 101.2280 | 1009.799 |
| W6a | 56208.60 | 63.226 | 17.759 | 11.291 | 5.294 | 1.561 | 4.603 | - | - | - | 23.480 | 4.565 | - | - | 108.660 | | 109.3838 | 96.57949 | 996.521 |
| W6b | 6.579 | - | 4.375 | - | - | - | - | - | - | - | - | 16.333 | 5.765 | 2.884 | - | | - | 300.8299 | 987.0926 |
| W7 | 1.917 | - | 1.167 | - | - | - | - | - | - | - | - | 16.614 | 4.861 | 1.329 | - | | - | 302.1385 | 985.9592 |
| W8 | 4.593 | 1.872 | 5.727 | 1.009 | - | 0.877 | - | 1.312 | - | - | 39.429 | 2.196 | - | - | - | | 63.95009 | 202.904 | 1032.097 |
| **Aver.**  **active**  **W2-W6** | 19697.070 | 189.345 | 20.838 | 29.427 | 4.634 | 3.765 | 11.066 | 1.387 | 0.614 | 0.476 | 27.371 | 6.352 | 2.860 | 0.299 | 363.673 | | 105.7691 | 127.7690 | 1031.5933 |
| **Aver.W1, W7 and W8**  **inactive** | 4.363 | 0.624 | 3.756 | 0.336 | - | 0.292 | - | 0.437 | - | - | 13.143 | 11.714 | 3.542 | 1.404 | - | | 21.31670 | 268.6242 | 1001.7163 |
| Upper Silesia - the Rymer Cones coal waste dump | | | | | | | | | | | | | | | | | | | |
| R1a | 4421.469 | 644.548 | 11.313 | 267.838 | 34.941 | 39.987 | 81.353 | - | 7.987 | 7.987 | 17.305 | 27.387 | 44.504 | 38.847 | 170.904 | 138.2679 | | 179.3061 | 969.4669 |
| R1b | 4537.745 | 331.575 | 41.324 | 118.125 | 60.114 | 17.106 | 27.305 | - | - | 5.01 | - | 6.442 | 6.442 | 3.847 | 140.462 | 197.0214 | | 132.9801 | 998.2689 |
| R2 | 89.084 | 34.688 | - | 58.74 | 10.009 | 13.824 | 26.266 | - | 2.669 | 4.004 | 32.029 | 13.729 | 29.174 | 18.448 | - | 9.6168 | | 289.6044 | 965.3662 |
| R3 | 643.748 | 49.669 | 6.424 | 17.719 | 9.393 | 2.595 | 5.189 | - | - | 0.726 | - | 1.288 | 1.16 | 0.693 | 60.018 | 26.69585 | | 269.7259 | 995.3178 |
| R4a | 12087.350 | 2142.488 | 85.152 | 997.172 | 230.125 | 144.003 | 313.952 | - | 50.096 | 45.087 | 21.291 | 101.461 | 175.544 | 128.885 | 244.781 | 291.5211 | | 39.06735 | 926.6858 |
| R4b | 719.652 | 80.545 | 6.261 | 29.531 | 10.351 | 4.355 | 7.784 | - | - | 1.102 | - | 3.221 | 3.221 | 0.616 | 44.361 | 21.89301 | | 277.1771 | 991.9029 |
| R5a | 8321.382 | 1418.501 | 30.795 | 624.257 | 89.448 | 89.603 | 188.709 | - | 19.659 | 18.349 | 13.106 | 60.672 | 102.806 | 80.523 | 167.377 | 177.9758 | | 137.8625 | 957.898 |
| R5b | 171.141 | 24.163 | 2.504 | 9.844 | 3.757 | 2.595 | 2.595 | - | - | 2.505 | - | 1.024 | 1.192 | 1.000 | 18.356 | 15.5037 | | 283.9983 | 991.3902 |
| R6a | 1042.594 | 1405.264 | 31.227 | 581.97 | 89.787 | 88.965 | 168.494 | - | 23.423 | 18.218 | 13.013 | 56.894 | 90.361 | 69.956 | 456.905 | 287.6025 | | 37.16045 | 964.3578 |
| R6b | 47.977 | 9.397 | 2.504 | 1.969 | 1.879 | - | - | - | - | 3.006 | - | 0.451 | 0.322 | 0.539 | 12.624 | 11.7825 | | 286.3668 | 992.0017 |
| **Aver.**  **all active** | 4146.548 | 614.084 | 21.750 | 270.716 | 53.980 | 40.303 | 82.165 | - | 20.767 | 10.599 | 19.349 | 27.257 | 45.472 | 34.335 | 131.579 | 117.7880 | | 193.3249 | 975.2656 |
| Upper Silesia - the Anna coal waste dump | | | | | | | | | | | | | | | | | | | |
| A1 | 1140.333 | 15.25 | 5.293 | 33.79 | 1.646 | - | - | - | - | 0.408 | 0.461 | - | - | - | 111.560 | 128.9506 | | 116.8241 | 991.6059 |
| A2 | 541.58 | 6.624 | 2.361 | 32.527 | 0.600 | - | - | - | - | - | 0.210 | - | - | - | 7.275 | 17.63346 | | 262.1281 | 925.4748 |
| A3 | 76.538 | 0.697 | 0.008 | 29.292 | - | - | - | 22.467 | - | - | 0.242 | - | - | 27.504 | 11.418 | 75.18087 | | 211.9934 | 970.7345 |
| A4 | 3052.96 | 71.477 | 0.904 | 65.888 | 0.712 | 0.796 | - | 21.717 | 1.193 | 1.179 | 1.536 | 2.255 | 1.309 | 66.364 | 223.018 | 93.27075 | | 197.2713 | 993.8549 |
| A5 | 13.014 | 0.121 | - | 37.481 | - | - | - | - | - | - | - | - | - | - | 9.719 | 82.10144 | | 205.5829 | 1017.99 |
| **Aver.**  **all active** | 964.885 | 18.834 | 1.728 | 39.796 | 0.592 | 0.159 | - | 8.837 | 0.238 | 0.317 | 0.490 | 0.451 | 0.262 | 18.774 | 72.598 | 79.42742 | | 198.7600 | 979.9321 |
| Upper Silesia - the Czerwionka Leszczyny coal waste dump | | | | | | | | | | | | | | | | | | | |
| CL1 | 2418.223 | 0.771 | 0.163 | 33.242 | - | - | - | 28.133 | - | - | - | - | - | 50.221 | - | 1.034179 | | 282.3568 | 923.4544 |
| CL2 | 191.594 | 51.322 | 0.784 | 36.013 | 0.317 | 1.944 | - | - | - | - | - | 0.544 | 0.145 | - | 4.814 | 23.60694 | | 251.8635 | 943.9478 |
| CL3 | 105.345 | 2.215 | 0.009 | 32.360 | - | - | - | - | - | - | - | - | - | - | - | 3.610146 | | 280.509 | 929.2107 |
| CL4 | 153.263 | 3.488 | 0.337 | 32.825 | 0.156 | 0.508 | - | - | - | - | - | - | - | - | 1.512 | 9.91059 | | 273.3547 | 933.5714 |
| CL5 | 100.769 | 1.051 | - | 16.069 | - | - | - | - | - | - | - | - | - | - | 0.257 | 8.152214 | | 275.4331 | 932.2339 |
| **Aver.**  **all active** | 593.839 | 11.769 | 0.275 | 30.102 | - | 0.490 | - | 5.626 | - | - | - | 0.109 | - | 10.044 | 1.317 | 9.262815 | | 272.7034 | 932.4836 |
| Lower Silesia - the Słupiec coal waste dump | | | | | | | | | | | | | | | | | | | |
| S1a | 1410.737 | 12.630 | - | 7.381 | 1.975 | 32.868 | 32.485 | 10.272 | 59.222 | 113.903 | 59.119 | 23.329 | 12.236 | 1.134 | - | 74.51772 | | 224.4055 | 1003.935 |
| S1b | 84.817 | 2.550 | - | 0.948 | - | 4.196 | 6.106 | - | 6.033 | 7.216 | 6.126 | 6.166 | 5.414 | 0.949 | - | 87.62723 | | 205.9139 | 1014.261 |
| S1c | 1860.638 | 1.649 | - | 0.446 | - | 3.223 | 5.008 | - | 4.826 | 7.697 | 5.365 | 6.125 | 3.513 | - | - | - | | 125.9404 | 175,9295 |
| S1d | 2232.013 | 310.993 | 35.846 | 62.891 | 23.521 | 9.725 | 31.471 | - | 8.633 | 11.017 | 6.989 | 8.778 | 11.233 | - | 48.447 | 182.6488 | | 84.44499 | 1053.9 |
| S1e | 3097.313 | 188.205 | 8.775 | 37.435 | 8.837 | 6.306 | 22.976 | - | 4.940 | 4.531 | 6.465 | 6.649 | 8.935 | 3.032 | - | 276.8319 | | 37.06365 | 1036.163 |
| S1f | 1898.299 | 119.839 | 4.003 | 22.064 | 7.062 | 4.933 | 12.965 | - | 2.930 | - | 4.207 | 4.988 | 4.849 | - | - | 239.0324 | | 41.95229 | 1058.127 |
| S1g | 1083.289 | 191.230 | 17.104 | 40.022 | 14.419 | 7.541 | 22.565 | - | 6.136 | 3.706 | 8.880 | 6.868 | 8.765 | - | 52.218 | 237.7143 | | 45.92019 | 1054.691 |
| S2a | 4195.581 | 208.973 | 24.674 | 41.750 | 18.234 | 10.786 | 2.969 | 4.813 | 10.974 | 3.872 | 10.602 | 11.766 | 12.601 | 3.869 | 8.502 | 249.8051 | | 47.80243 | 1041.878 |
| S2b | 6286.977 | 238.133 | 22.461 | 35.312 | 21.179 | 11.110 | 32.700 | 0.890 | 13.063 | 4.447 | 13.272 | 11.530 | 13.330 | 4.177 | - | 260.5868 | | 39.74778 | 1038.496 |
| S3 | 4001.632 | 260.327 | 20.197 | 55.181 | 20.665 | 9.211 | 35.157 | - | 11.408 | 6.143 | 14.212 | 9.209 | 13.513 | 3.451 | 55.832 | 264.3131 | | 35.37275 | 1043.14 |
| S4a | 0884.594 | 98.925 | 0.823 | 40.969 | 2.615 | 25.136 | 40.049 | - | - | - | 2.961 | 26.178 | 16.677 | 7.213 | - | 196.8504 | | 62.66983 | 1068.587 |
| S4b | 1663.305 | 131.377 | 3.995 | 32.683 | 5.912 | 8.812 | 22.023 | - | 2.822 | - | 5.334 | 7.499 | 7.436 | 2.861 | 173.413 | 234.8074 | | 51.8610 | 1050.117 |
| S5a | 3253.376 | 19.899 | - | 25.578 | 1.356 | 2.547 | 41.017 | - | - | - | 3.236 | 26.150 | 20.329 | 7.787 | - | 204.6261 | | 80.35486 | 1044.105 |
| S6 | 1699.935 | - | - | 0.469 | - | - | - | - | - | - | - | - | - | - | - | 42.95211 | | 254.5318 | 997.3391 |
| S7 | 3582.071 | 3.261 | - | 1.284 | 0.586 | 1.091 | - | - | - | - | 8.812 | - | - | - | - | 227.5923 | | 55.7773 | 1050.508 |
| S8a | 737.481 | 131.708 | 2.099 | 28.026 | 6.860 | 4.996 | 14.111 | - | 3.639 | - | 5.273 | 4.059 | 5.235 | - | 140.36 | 185.8915 | | 61.92654 | 1076.282 |
| S8b | 1335.053 | 131.355 | 2.656 | 29.784 | 7.884 | 5.291 | 18.172 | - | 6.223 | 2.024 | 6.933 | 5.116 | 7.237 | 2.818 | 21.521 | 221.6609 | | 42.31893 | 1069.524 |
| S9 | 1038.287 | 115.659 | 9.566 | 24.469 | 4.327 | 5.766 | 15.053 | - | - | - | 26.870 | 7.783 | 7.868 | 3.304 | 12.682 | 185.5874 | | 59.75556 | 1077.868 |
| S10a | 86.904 | 4.333 | - | 0.648 | - | - | - | - | - | - | - | - | - | - | - | 179.4167 | | 136.0736 | 1016.99 |
| S10b | 12.797 | 22.555 | 15.625 | - | - | - | - | 16.552 | - | - | - | - | - | - | - | 4.277191 | | 303.0562 | 982.3703 |
| S11 | 2.396 | 1.094 | 2.491 | - | - | - | - | - | - | - | - | 13.208 | 6.168 | 1.722 | - | - | | 302.8821 | 985.3059 |
| **Aver.**  **S1-S10**  **active** | 1933.464 | 109.725 | 8.391 | 24.392 | 7.272 | 8.726 | 17.796 | 0.626 | 7.103 | 8.204 | 9.771 | 8.612 | 8.054 | 2.077 | 2.9333 | 182.2183 | | 103.8432 | 1039.627 |
| **S11**  **inactive** | 2.396 | 1.094 | 2.491 | - | - | - | - | - | - | - | - | 13.208 | 6.168 | 1.722 | - | - | | 302.8821 | 985.3059 |
| Lower Silesia - the Nowa Ruda and Przygórze coal waste dumps | | | | | | | | | | | | | | | | | | | |
| N1 | 54.952 | 1.015 | 18.418 | 2.952 | 0.340 | 27.369 | 16.073 | - | 29.463 | 64.209 | 32.567 | 43.916 | 19.438 | 2.372 | - | - | | 294.1271 | 982.0199 |
| N2 | 115.284 | 0.599 | 23.837 | 0.229 | - | 0.404 | 0.576 | - | - | - | 11.508 | 11.858 | 4.690 | 4.454 | - | 32.34755 | | 258.0063 | 983.9585 |
| N3 | 142.819 | 1.628 | 20.863 | 0.233 | - | 0.227 | 0.555 | - | - | - | 18.386 | 11.373 | 4.248 | 3.841 | 12.555 | 39.25923 | | 256.1175 | 991.7994 |
| P1 | 50.501 | - | 24.140 | 0.196 | - | 0.303 | 0.585 | - | - | - | 15.000 | 11.921 | 4.393 | 4.429 | - | 5.9408 | | 290.9820 | 980.3309 |
| **Aver.**  **active N2, N3** | 129.051 | 1.114 | 22.350 | 0.231 | - | 0.315 | 0.565 | - | - | - | 14.947 | 11.616 | 4.469 | 4.148 | 6.277 | 35.80339 | | 257.0619 | 987.8790 |
| **aver. inactive**  **N1, P1** | 52.726 | 0.507 | 21.279 | 1.574 | 0.170 | 13.836 | 8.329 | - | 14.731 | 32.104 | 23.783 | 27.918 | 11.915 | 3.400 | - | 2.970400 | | 292.5545 | 981.1754 |
| **US aver. active** | 6350.585 | 208.508 | 11.148 | 92.510 | 14.825 | 11.180 | 23.308 | 3.963 | 5.405 | 2.848 | 11.802 | 8.542 | 12.156 | 15.863 | 142.292 | 78.06184 | | 198.1393 | 979.8187 |
| **US aver. inactive** | 4.363 | 0.624 | 3.756 | 0.336 | - | 0.292 | - | 0.437 | - | - | 13.143 | 11.714 | 3.542 | 1.404 | - | 21.31670 | | 268.6242 | 1001.7160 |
| **LS aver. active** | 1031.258 | 55.419 | 15.371 | 12.312 | 3.636 | 4.520 | 9.181 | 0.813 | 3.551 | 4.102 | 12.359 | 10.114 | 6.261 | 3.112 | 17.805 | 109.01090 | | 180.4525 | 1013.7530 |
| **LS aver. inactive** | 51.613 | 0.254 | 22.709 | 0.885 | 0.085 | 7.069 | 4.457 | - | 7.366 | 16.052 | 19.392 | 19.920 | 8.154 | 3.914 | - | 4.45560 | | 291.7683 | 980.75310 |
